# Inflammasome-Mediated Inflammation in Liver Ischemia-Reperfusion Injury

**DOI:** 10.3390/cells8101131

**Published:** 2019-09-23

**Authors:** Mónica B. Jiménez-Castro, María Eugenia Cornide-Petronio, Jordi Gracia-Sancho, Carmen Peralta

**Affiliations:** 1 Institut d’Investigacions Biomèdiques August Pi I Sunyer (IDIBAPS), 08036 Barcelona, Spain; monicabjimenez@hotmail.com (M.B.J.-C.); cornide@clinic.cat (M.E.C.-P.); 2 Liver Vascular Biology Research Group, Barcelona Hepatic Hemodynamic Laboratory IDIBAPS, 08036 Barcelona, Spain; jordi.gracia@idibaps.org; 3 Centro de Investigación Biomédica en Red de Enfermedades Hepáticas y Digestivas (CIBERehd), 08036 Barcelona, Spain

**Keywords:** inflammasome, liver, ischemia-reperfusion injury, partial hepatectomy, transplantation, steatosis

## Abstract

Ischemia-reperfusion injury is an important cause of liver damage occurring during surgical procedures including hepatic resection and liver transplantation, and represents the main underlying cause of graft dysfunction and liver failure post-transplantation. To date, ischemia-reperfusion injury is an unsolved problem in clinical practice. In this context, inflammasome activation, recently described during ischemia-reperfusion injury, might be a potential therapeutic target to mitigate the clinical problems associated with liver transplantation and hepatic resections. The present review aims to summarize the current knowledge in inflammasome-mediated inflammation, describing the experimental models used to understand the molecular mechanisms of inflammasome in liver ischemia-reperfusion injury. In addition, a clear distinction between steatotic and non-steatotic livers and between warm and cold ischemia-reperfusion injury will be discussed. Finally, the most updated therapeutic strategies, as well as some of the scientific controversies in the field will be described. Such information may be useful to guide the design of better experimental models, as well as the effective therapeutic strategies in liver surgery and transplantation that can succeed in achieving its clinical application.

## 1. Ischemia-Reperfusion Injury, an Unresolved Problem in Clinical Practice

Ischemia-reperfusion (I/R) injury is a two-stage phenomenon in reduced blood flow to an organ, resulting in hypoxia, causes cell damage, which is then exacerbated upon restoration of oxygen delivery [1]. Hepatic I/R injury, an inherent phenomenon in liver resection or liver transplantation (LT) is associated with post-operative morbidity and mortality [2]. Adverse outcomes due to hepatic I/R injury persist as a major unresolved problem in clinical practice. The mechanisms responsible are extremely complex, involving a great number of cellular components, factors and mediators (reactive oxygen species (ROS), neutrophil infiltration, and microcirculatory dysfunction, among others) [1,2,3,4,5,6]. To date, research data have produced several controversies, and even discrepancies, in our understanding of this pathology. Of particular interest, studies suggest that the type (cold or warm), extent (partial or total) and duration/timing of ischemia (from minutes to hours) of ischemia, together with the occurrence of liver regeneration (associated with liver resection), may all alter the mechanisms of liver I/R injury and regenerative failure as well as the effects of the strategies analyzed to date [7].

Hepatic I/R can result from both warm and cold ischemia, which must be distinguished because of existing controversy about the comparative pathophysiological mechanisms of each category. Whereas, warm I/R is observed in vascular occlusion associated with hepatic resection, trauma or hemorrhagic shock, cold I/R is evident during LT, where the graft is subjected to cold ischemia prior to implantation in the recipient [8]. The main cell types affected by ischemic injury are the hepatocytes and the liver sinusoidal endothelial cells. Hepatocytes and liver sinusoidal endothelial cells are more sensitive to warm and cold ischemia, respectively [9,10,11]. In warm ischemia, the lack of O_2_ in hepatocytes interrupts the electron flow causing the respiratory chain to become reduced and leads to cellular adenosine triphosphate (ATP) depletion, acceleration of anaerobic glycolysis, increased lactate generation, and alterations in H^+^, Na^+^, and Ca^2+^ homeostasis, which, together, inflict serious damaging effects on the hepatocyte [12,13]. Reperfusion injury derives mainly from toxic ROS generated upon the reintroduction of O_2_ to ischemic tissues. ROS are produced from both intracellular and extracellular sources, with the mitochondria being their major source in liver cells [14]. Conversely, most hepatocytes remain viable after 48 h of cold ischemia, however the liver sinusoidal endothelial cells suffer severe damage following reperfusion [15,16]. The result of this sinusoidal damage is subsequent microcirculatory disorders and hepatic dysfunctions following reperfusion, which contribute to the development of primary nonfunction or impaired primary function after LT [10,17]. In addition, alterations in hepatocyte levels of adenine nucleotides during cold ischemia can trigger proteolytic events that compromise liver graft functions after LT [18]. Moreover, cold ischemia disturbs several key hepatocellular functions, such as pH homeostasis, which contribute to preservation injury of the liver graft [7].

Another variable factor to be characterized in I/R injury is the percentage of hepatic ischemia applied. The severity of hepatic injury as well as the hepatic I/R mechanisms, such as the recovery of blood flow and energy charge during reperfusion, are dependent on the extent of ischemia i.e., whether total or partial hepatic ischemia is applied [19]. This differentiation can be described by reference to the stealing phenomenon. In contrast to 100% hepatic ischemia, during 70% ischemia (ischemia in the left and median hepatic lobes), the flow is shunted via the right lobes and following the release of the occlusion of the left and median lobes, an amount of shunting via the right lobes will continue during hepatic reperfusion until vascular resistance in the post-ischemic lobes decreases. Thus, the recovery of blood flow of the pre-ischemic lobe is later in partial (70%) than in total hepatic ischemia [20]. Conversely, total compression of the hepatoduodenal ligament in total ischemia increases the risk of liver ischemic lesions and intestinal congestion; a risk factor which is absent in partial hepatic ischemia [21]. It is therefore, clear that different mechanisms contribute to hepatic damage, depending on the percentage of hepatic ischemia. Thus, in line with these observations, the protective effects of some drugs are dependent on the extent of hepatic ischemia applied [22,23].

The literature draws on research data that support the differential effects of ischemia in hepatic I/R injury, according to the timings. In particular, it has been observed that the severity of hepatocyte damage differs, depending on the duration (timing) of ischemia. It has been reported that 60 min of warm ischemia results in reversible cell injury since liver oxygen consumption returns to control levels when oxygen is resupplied following reperfusion. However, reperfusion after more prolonged periods of warm ischemia (120–180 min) results in irreversible cell damage. These observations accord with a previous report on hepatic I/R, indicating a cellular endpoint for hepatocytes after 90 min of ischemia [24]. As with the degree of hepatic damage, the mechanisms responsible for hepatic I/R are different depending of the duration of ischemia. Thus, experimental models of partial hepatic I/R (30–45 min ischemia) have evidenced a minor role of inducible nitric oxide synthase in damage [25], while with ischemia of 60 min or longer, changes in the expression of inducible nitric oxide synthase become relevant for liver I/R injury [26]. 

In human hepatic resections with a warm ischemia lasting less than 75 min, subsequent liver function was only mildly impaired. However, after prolonged ischemia, hepatic functions are compromised, especially in non-healthy livers such as those exhibiting the characteristic of steatosis [27,28]. In line with this, in human LT a long ischemic period is a predictor for post-transplantation graft dysfunction, and some transplantation centers hesitate to transplant liver grafts that have been preserved for more than 10 h. However, primary organ dysfunction can also result from LT following shorter ischemic periods. In LT following cold ischemia, the mechanisms responsible for hepatic I/R are, similarly, dependent on its duration [29]. Thus, in the experimental model of LT, xanthine dehydrogenase/xanthine oxidase (ROS generation system) plays a crucial role in I/R injury at 16 h of cold ischemia, conditions under which significant conversion of xanthine dehydrogenase to xanthine oxidase occurs (80–90% of xanthine oxidase). However, xanthine dehydrogenase/xanthine oxidase does not appear to be crucial in shorter cold ischemic periods, such as 6 h [29]. Moreover, oxidative stress in hepatocytes and the stimulatory state of Kupffer cells after I/R also differ, dependent on the duration of ischemia. In sum, the mechanisms of liver damage and consequently the design of specific strategies for protecting against liver I/R injury, are dependent on the duration of ischemia.

The mechanisms responsible for hepatic I/R are dependent on the condition of the liver (steatotic versus non-steatotic ones). Hepatic steatosis is defined as lipid accumulation in hepatocytes and occurs frequently in both cadaveric and living donors (reported in between 9% and 26% of donors) [30,31,32]. Hepatic steatosis is a major risk factor for liver surgery since steatotic livers show comparatively impaired regenerative responses and reduced tolerance to I/R injury. Hence, steatosis is associated with an increased complication index and post-operative mortality after major liver resection and LT. Unilateral and combined causes of hepatic steatosis include obesity, older age and alcoholism [33]. Two types of hepatic steatosis have been described: (1) Macrovesicular steatosis, in which the fat vacuoles occupy most of the hepatocytes cytoplasm and displace the nucleus peripherally; (2) Microvesicular steatosis, where the vacuoles are smaller and have a centrilobular distribution. The intracytoplasmic fat droplets increase hepatocellular volume, which induces distortion and narrowing of the sinusoids and causes alterations in liver microcirculation [34]. Severity of steatosis is graded as mild < 30%, moderate 30–60%, and severe > 60%.

Livers with macrovesicular steatosis are more intolerant to ischemic damage than those with microvesicular steatosis [35]. Thus, transplantation outcomes are not affected by hepatic microvesicular steatosis, regardless of its severity. In macrovesicular steatosis, the use of grafts with moderate steatosis is controversial because some reports have shown an association with increased incidence of primary nonfunction following LT [7] whereas, other authors have reported excellent results [36]. Severe macrovesicular steatosis precludes organs from being used in transplantation because of a high risk of graft failure [35]. Indeed, some highly vigilant transplant programs even exclude donors with mild macrovesicular steatosis [37]. Given the wide variety of observations reported, we consider transplant dysfunction to be multifactorial, meaning that graft steatosis cannot be isolated as the only cause of dysfunction or initial nonfunction after LT [38]. In fact, a large study (5051 patients) showed that when cold ischemia extends beyond 11 h, macrovesicular steatosis for only 20% is associated with an increased risk of graft loss [39]. Thus, organs with >30% steatosis are used only if other known risk factors are controlled i.e., donor age < 40 years, short cold ischemia time of <5 h, and non-circulatory cause of death [37,40].

The presence of steatosis is associated with an increased mortality risk between 2% and 14%, following liver resection surgery [41,42]. Indeed, all grades of steatosis significantly predicted post-operative complications. Among patients with hepatic steatosis, cholestasis was a significant risk factor for mortality after liver resection [41]. Clinical studies indicate that livers with mild to moderate hepatic steatosis is associated with exacerbated damage when compared to healthy livers [43]. Clinical studies of 135 liver surgery patients indicated mortality rates of 7% and 14% in those with mild steatosis (<30%) and moderate to severe steatosis (>30%), respectively [44]. In addition, those with moderate to severe steatosis suffered more from post-operative hepatic dysfunction. Different studies, including a meta-analysis of 1000 patients, revealed a twofold increase in post-operative morbidity rates among steatotic patients and an almost threefold risk of mortality for those with the severest form of steatosis, following hepatectomy [45,46]. In a study of 2715 patients, 927 of whom presented with steatosis, Hamady et al. found that patients with liver steatosis run a substantially higher risk of post-operative liver failure [47]. These studies confirmed separate reports of increased morbidity associated with steatosis in patients undergoing major hepatic resection and further, that patients with macrosteatosis had increased mortality and morbidity incidences, over those with microsteatosis. Contrarily, no statistically significant increase in postoperative complications could be observed following major hepatectomy in obese steatotic patients compared with matched non-obese, non-steatotic controls [48,49]. The largest single-center review of liver surgery outcomes was reported in 2002, by Jarnagin et al., who looked at 1803 hepatic resections. Steatosis was identified in 325 patients and, in contrast to other reports, did not affect post-operative outcomes. The authors attributed their different results to the relatively small number of steatotic patients compared to those with healthy livers, in addition to difficulties in optimizing techniques for the evaluation of hepatic steatosis [50].

The inflammation inherently associated with liver I/R is exacerbated by the presence of steatosis. Oxidative stress, impairment of microcirculation, neutrophils and endoplasmic reticulum stress have been implicated in the increased susceptibility of steatotic livers to I/R injury [51,52]. The angiotensin-converting enzyme inhibitor–Angiotensin II–angiotensin II receptor and angiotensin-converting enzyme 2–Angiotensin–(1-7)–Mas receptor axis play a major role in non-steatotic and steatotic grafts, respectively [53]. We observed that, compared with non-steatotic liver, the presence of steatosis resulted in reduced retinol-binding protein 4 and toll-like receptor 4 (TLR4) levels and increased peroxisome proliferator-activated receptor γ levels [54,55]. The vulnerability of steatotic livers subjected to ischemia is also associated with reduced adiponectin and resistin levels [56]. In contrast to responsive programmed apoptosis observed in non-steatotic livers following I/R injury, it has been reported that hepatocytes with fatty infiltration developed massive necrosis. This may be due to low ATP production and dysfunction among the regulators of apoptosis [57].

Moreover, in addition to damage and regenerative failure in the liver, I/R injury also induces a systemic inflammatory response in extra-hepatic organs. Both the anhepatic phase of LT and the vascular occlusion used in hepatic resection result in splanchnic congestion, affecting the intestine and lungs, among other organs [58,59,60,61,62]. It is, therefore, clearly of clinical and scientific importance to develop protective strategies in liver surgery [63].

## 2. Inflammasome and Its Implications in Liver Disease

The term inflammasome refers to large intracellular multi-protein complexes that detect intracellular danger and respond to pathogenic and other threats [64]. Complexes are characterized by three main components: (a) an intracellular receptor, such as nucleotide-binding oligomerization domain (NOD)-like receptors (NLR) or absent in melanoma 2 (AIM2)–like receptors; (b) the apoptosis-associated speck-like protein containing a caspase recruitment domain (ASC) that links two other components together, an amino-terminal pyrin domain and a carboxy-terminal caspase recruitment domain (CARD); and (c) an effector protein that is often pro-caspase-1 [65,66,67,68,69].

**Figure 1:**
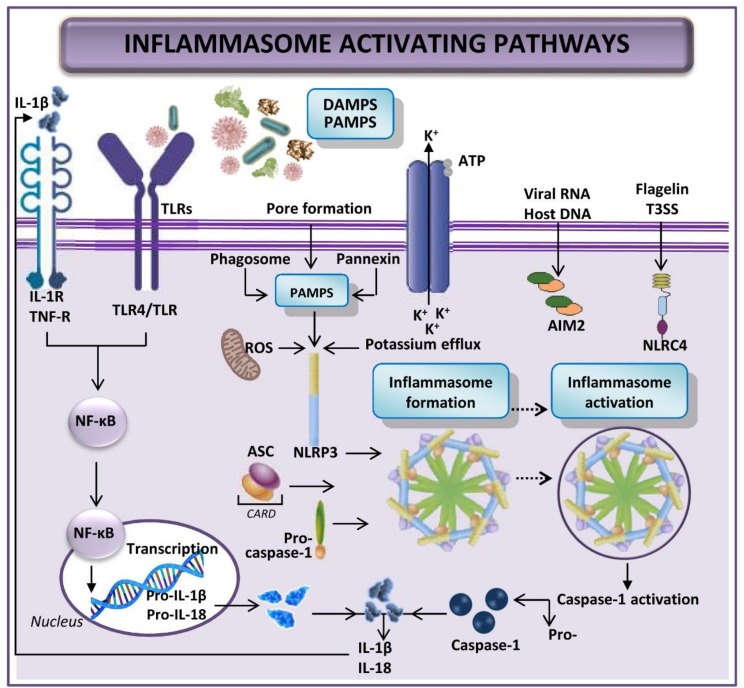
Schematic representation of the inflammasome activation pathways. Firstly, expression of inflammasome components is activated. Secondly, signaling by DAMPs and PAMPs results in the inflammasome activation, which entails pro-caspase-1 activation and cleavage of pro-IL-1β and pro-IL-18 into their mature forms. Abbreviations: AIM2, absent in melanoma 2; ASC, Apoptosis-associated speck-like protein containing a caspase recruitment domain (CARD); ATP, Adenosine triphosphate; DAMP, damage-associated molecular pattern; IL, Interleukin; IL-1R, Interleukin-1 receptor; NFκB, nuclear factor kappa B; NLRC4, NLR family CARD domain-containing protein 4; NLRP3, NLR pyrin domain containing protein 3; PAMP, pathogen-associated molecular pattern; ROS, reactive oxygen species; T3SS, type III secretion system; TLR, Toll-like receptor; TNF, Tumor necrosis factor.

Since inflammasome activation induces an inflammatory response, it must be tightly regulated. With a few exceptions, activation of most inflammasomes is thought to be a two-step process: firstly, TLR or interleukin-1 receptor (IL-1R) signaling triggers the expression of inflammasome components (NLRs, ASC, pro-caspase-1, pro-interleukin-(IL)-1β, and pro-IL-18); secondly, damage-associated molecular patterns (DAMPs)- and pathogen-associated molecular patterns (PAMPs)-mediated signaling initiates production of the multi-protein NLR inflammasome, which entails pro-caspase-1 activation and cleavage of pro-IL-1β and pro-IL-18 into their mature forms [70,71]. Secretion of these cytokines causes immune cells, especially neutrophils and macrophages, to intervene, instigating inflammation of damaged tissue (Figure 1) [72,73,74]. IL-1β may further activate the IL-1β-receptor leading to amplification of inflammasome signaling [70,71]. In addition, affected tissue undergoes both apoptosis and another inflammation-related cell death named pyroptosis (a rapid inflammatory form of lytic programmed cell death), after the inflammasome activation [72,74,75,76].

A recent publication suggests that the priming step is required only for activation of NLR pyrin domain containing protein 3 (NLRP3), not the other inflammasomes such as NLR-family CARD domain containing protein 4 (NLRC4) or AIM2 [77]. In NLRP3, the first step of inflammasome construction prepares the cell, and it is only after the recognition of an NLRP3 activator that NLRP3 is fully activated and inflammasome formed. Although most pattern recognition receptors have limited specificity for one or a few related PAMPs or DAMPs, NLRP3 is unique in that it is activated by a wide variety of unrelated stimuli, including bacterial and viral infections, as well as by sterile inflammation mediated by endogenous DAMPs [69]. All these activators induce cellular stress; cellular stress is then sensed by NLRP3. However, specific elucidation of the mechanisms by which NLRP3 senses cellular stress and which pathways are marshalled to culminate in full NLRP3 activation and inflammasome formation has yet to be articulated [69].

The function of AIM2 in the inflammasome is sensing cytosolic dsDNA [78,79,80]. It is activated by bacterial, viral or host DNA and can directly bind to its ligand, thus potentially being implicated in the pathogenesis of autoimmune diseases by recognizing the mammalian DNA [78,79,80,81]. Signaling triggers caspase-1 activation [78,79,80]. Caspase-1 activation can also occur as a result of inflammasome activation by dsRNA via the helicase receptor RIG-I, after association with the inflammasome adaptor molecule ASC [77,82].

NLRC4 inflammasome is activated by the flagellin of Gram-negative and Gram-positive bacteria or the type III secretion system of Gram-negative bacteria [83,84,85]. Zhao et al. have reported other NLR proteins, such as murine NAIP5 and NAIP2, interacting with the bacterial flagellin and the type III secretion system rod components, respectively, to promote NLRC4 activation and inflammasome formation [86]. Certainly, the type III secretion system needle subunit is recognized by human NAIP [77,86]. However, further exploration is needed to plot the individual and related processes leading to NRLC4 inflammasome activation.

It is known that various types of inflammasomes, such as NLRP1, NLRP2, NLRP3, NLRP6, NLRP10, and NLRP12, have been expressed in different hepatic diseases [64,72]. DAMPs and PAMPs can trigger the activation of inflammation in hepatic I/R [70,72,87,88]. We do not yet fully understand the specific context in which DAMPs activate inflammasome, however, it seems that the abundance of each DAMP is dependent on the type of injury inflicted to the hepatocytes [70]. DAMPs have been widely evaluated for their role in hepatic I/R and are the best-characterized inflammasome-activating signals in liver disease. They are mostly derived from damaged hepatocytes and include ATP, uric acid, cholesterol crystals, palmitic acid, DNA fragments, fatty acids and ROS. In contrast, PAMPs are mostly derived from the gut, due to gut alterations microbiota composition and increased permeability during splanchnic congestion. But, studies have yet to elucidate the role of PAMPs in hepatic I/R [64,70,88].

Studies of liver disease may be related to individual risk factors or combinations of different pathogens and the immune response to the presence of threat. Among these, NLRP3 inflammasome has been implicated in both alcoholic and non-alcoholic steatohepatitis. Chronic administration of ethanol in mice increased hepatic levels of IL-1β, pro-caspase-1, ASC and NLRP3 [88]. Hepatic mRNA levels for NLRP3, ASC, and caspase-1 are up-regulated in experimental models of non-alcoholic fatty liver disease, but full activation of inflammasome has only been evidenced in non-alcoholic steatohepatitis [89]. In patients with alcoholic liver disease or non-alcoholic steatohepatitis, increased hepatic levels of inflammasome components together with increased serum levels of IL-1β have been reported and associated with liver damage in alcoholic liver disease [70,90,91]. Inflammasome activation and IL-1β generation have also been correlated with liver fibrosis [92]. Accordingly, constitutive activation of NLRP3 activated hepatic stellate cell and increased collagen deposition. In hepatitis B, elevated hepatic expression of the inflammasome AIM2 correlates inflammation and with the expression of caspase-1, IL-1β and IL-18 [70,93]. Hepatic damage induced by paracetamol overdose has been linked to inflammasome activation, as mice deficient in NLRP3, caspase-1, or ASC are protected against damage [94]. Down-regulation of the components of NLRP3 inflammasome in patients with hepatocellular carcinoma (HCC) correlates with advanced stages [95]. Conversely, luteoloside suppressed metastasis of HCC cells via NLRP3 inflammasome inhibition [70,96].

Only a limited number of treatments such as caspase inhibitors, and IL 1β action inhibitors are available for liver diseases [64]. IDN-6556173 and PF-03491390174 (pan-caspase inhibitors) have been evaluated in human liver diseases. In LT, the addition of IDN-6556 in preservation solution protected liver grafts against I/R damage and inflammation. Furthermore, in patients infected with hepatitis C virus, IDN-6556 and PF-03491390 decreased hepatic damage, with no reduction in hepatitis C virus viral load. However, inflammasome components were not studied in such clinical studies based on LT [97,98,99]. In an experimental model of alcoholic liver disease, anakinra (an IL-1R antagonist (IL-1RA)) attenuated liver steatosis, inflammation, damage and fibrosis [64]. Some drugs already in clinical use can inhibit inflammasome activation, including glyburide (used for type 2 diabetes mellitus), a specific inhibitor of the NLRP3 inflammasome and probenecid, a P2 × 7 receptor inhibitor. The diarylsulfonylurea compound Mcc950 has been considered the most potent and specific NLRP3 inhibitor. Mcc950 specifically inhibits NLRP3 inflammasome activation in preclinical liver diseases, including steatohepatitis but a phase II clinical trial of Mcc950 for rheumatoid arthritis was suspended owing to hepatic toxicity [69,100].

## 3. Relevance of Inflammasome in Hepatic Ischemia-Reperfusion Injury

To gain better understanding of the pathophysiology of hepatis I/R, leading to the development of more effective treatment and improved surgery outcomes, one focus of study could be the associated activation of inflammasomes. The inflammasome plays an important role in the pathogenesis of liver I/R injury and has been identified as a major contributor to hepatocyte damage, immune cell activation and amplification of liver inflammation [64,72]. In our view, examination of this role can contribute to new therapeutic strategies and/or biomarkers in the field of liver diseases.

The present review aims to summarize the role of inflammasome in the pathogenesis of hepatic I/R injury. Moreover, we highlight the mechanisms involved in activation of the complex with a clear distinction between steatotic and non-steatotic livers and between warm and cold I/R. Finally, we describe the most up to date therapeutic strategies as well as some of the scientific controversies in the field. This may be useful to guide the design of better experimental models and transfer the benefits of successful therapeutic interventions into clinical application in liver surgery.

### 3.1. Inflammasome in Warm Ischemia-Reperfusion Associated with Liver Resection

#### 3.1.1. Role of Inflammasome in Experimental Models of Warm Ischemia-Reperfusion Injury without Hepatic Resection

In 2011, Zhu et al. discovered that caspase-1 and NLRP3 are increased, in an experimental model of partial (70%) warm ischemia for 60 min in mice. Utilizing advances in molecular biology, that provide novel gene therapy options for experimental treatments in liver disease and hepatic surgery, they were able, in addition, to observe that gene silencing of NLRP3 via shRNA plasmid suppressed the activation of hepatic caspase-1, protecting against I/R injury [101,102]. This gene therapy was associated with reduced production of the pro-inflammatory cytokines IL-1β, IL-18, TNF-α, and IL-6; reduced production of DAMP (HMGB1, an early mediator of injury and inflammation in liver I/R) and reduced inflammatory cell infiltration [101]. Further uses of gene therapy techniques include, for instance, suppression of the ROS burst by superoxide dismutase and catalase transfection by either adenovirus, liposomes or polyethyleneglycol [3,103,104]. To inhibit apoptosis, overexpression of Bag-1 and Bcl-2, mainly by using adenovirus has been tested [3]. To limit neutrophil activation, reduction in ICAM-1 expression was obtained by using liposomes [105]. Cytoprotective strategies based on the expression of heme oxygenase-1 (HO-1), anti-inflammatory cytokine IL-13 and IL-1RA and inhibition of IκBalpha have been developed employing adenoviral or liposome vector [103,104,106,107,108,109]. However, given the experimental data, there are a number of problems inherent in gene therapy, such as vector toxicity and difficulties in increasing appropriate transfection efficiencies [110].

Interestingly, Inoue et al., also found that partial (70%) hepatic warm ischemia for 60 min in mice up-regulated NLRP3, but not ASC [111]. Therefore, NLRP3−/− mice, but not ASC−/− and caspase-1−/− mice, showed reduced inflammatory responses and apoptosis following hepatic I/R. NLRP3-/- mice, but not ASC−/− induced changes in IL-1β. In addition, authors also observed that hepatic I/R injury was attenuated in IL-1β−/− mice. Moreover, authors suggested that NLRP3 regulates inflammatory response including IL-1β levels and recruitment of neutrophils in hepatic I/R independently of the inflammasome [111]. Contrarily, in an experimental model of partial (70%) warm ischemia for 90 min, Kamo et al. found that ASC-deficient mice showed caspase-1/IL-1β signaling suppression, leading to protection against liver I/R damage. This was evidenced by enhancement of anti-apoptotic functions, and down-regulation of the HMGB1-TLR4-pathway [112]. The discrepancy between the studies regarding to the role of ASC and IL-1β could be a consequence of the different hepatic I/R protocol used and the extent of injury. Compared with 60 min of ischemia, 90 min induced excessive inflammation and injury in the liver [111,112]. In addition, it should be noted that timing of ischemia may all alter the mechanisms of liver I/R injury. Indeed, the role of IL-1β in hepatic I/R injury is controversial. In a model of partial (70%) warm ischemia for 90 min, Kato et al. found that there was no difference in hepatic injury between wild-type and IL-1R–deficient mice and suggested a limited role of IL-1β in hepatic I/R injury [113]. This is of scientific interest since in the study reported by Kamo et al., [112] the relevance of the changes in IL-1β induced in ASC-deficient mice was not evaluated. Moreover, the regulation of IL-1β actions on hepatic I/R damage was not determined in such conditions. Further investigation of this issue is therefore needed.

Conversely, other studies have shown up-regulation of hepatic IL-1β in I/R, after partial (70%) warm ischemia for 60 min. Mice deficient in IL-1R1 or treated with the IL-1R antagonists showed reduction in hepatic I/R injury, liver inflammation, and neutrophil infiltration [107,114,115,116]. These studies did not investigate the potential relationship between inflammasome and IL-1β. However, the effects on damage indicated by the regulation of IL-1β actions are similar to those described by Inoue et al. In evaluating the potential relationship between inflammasomme and IL-1β, their results indicated that hepatic I/R injury was attenuated in IL-1β−/− mice, and that the inflammasome-independent, IL-1β–driven inflammatory responses appear to be important in hepatic I/R injury [111]. The roles of different components of inflammasome in hepatic I/R would seem, therefore, to differ, according to the percentage and timing of ischemia, requiring specific drugs for each type of intervention, to regulate the role of inflammasome.

To evaluate the mechanisms involved in inflammasome activation, different knockout and mutant mice were submitted to partial (70%) hepatic warm ischemia for 60 min [117]. Authors found that during liver I/R, the NLRP3 inflammasome is activated in Kupffer cells by endogenous extracellular histones through a TLR9-dependent pathway. Neutrophils and monocytes are thereby recruited, increasing damage in the liver [117]. Authors provide evidences that the administration of exogenous histones can activate the NLRP3 inflammasome in NLRP3-KO mice subjected to 70% of hepatic warm ischemia for 60 min, whereas anti-histone antibody treatment, which neutralizes endogenously released histones, reduced the activation of NLRP3 in wild-type mice during liver I/R. Furthermore, NLRP3 activation was reduced when TLR9 antagonist was administrated jointly with exogenous histones, suggesting that NLRP3 activation by extracellular histones is dependent on the TLR9-signaling pathway. This requires more research, as we do not yet understand fully the mechanism of uptake and delivery of histones and DNA to TLR9 [117]. In addition to the TLR9-dependent pathway, both pannexin-1 and cathepsin B are required for inflammasome activation in Kupffer cells in hepatic I/R. ATP and crystalline material, such as uric acid or cholesterol are implicated in this process [118]. Thus, treatment with pannexin-1 inhibitor and anti-cathepsin B antibody, or pannexin-1 and cathepsin B gene silencing, attenuated I/R-induced inflammasome activation and hepatic injury after 60 min of partial (70%) warm ischemia. Moreover, authors showed that treatment with the antioxidant *N*-acetylcysteine reduced pannexin-1 protein expression and cathepsin B release, and the depletion of Kupffer cells with gadolinium chloride decreased the expression of NLRP3 and AIM2 inflammasome and the activation of their signaling pathways [118]. However, we should be cautious regarding the evaluation of such non-specific drug interventions in the regulation of inflammasome. Effects of concomitant administration of such drugs (i.e., pannexin, cathepsin) and inflammasome regulators needs further study.

Studies have shown that X-box-binding protein 1 signaling pathway was modulated by the heat shock transcription factor 1 (HSF1)- β-catenin axis, thus regulating NLRP3 behavior [119]. In fact, results from myeloid-specific HSF1 knockout (HSF1M-KO) mice, submitted to 90 min of partial (70%) warm ischemia, indicate that HSF1 activation is required to promote β-catenin signaling, which, in turn, inhibits X-box-binding protein 1, leading to NLRP3 inactivation and reduced liver I/R injury [119]. Future investigation will be required to evaluate the extent to which the damage limitation benefits of the (HSF1)-β-catenin axis are delivered through NLRP3 inactivation. Based on the emerging function of HSF1 in protection against oxidative stress-induced injury, it is becoming clear that HSF1-β-catenin signaling is a key player in the regulation of immunity during liver I/R injury. The development of HSF1 activators could be a potential intervention for treatment of I/R-induced liver inflammation [119]. Nevertheless, it remains to be seen whether these results, obtained at 90 min of hepatic ischemia, will be mirrored in procedures involving up to 60 min of ischemia, as is often the case in hepatic resections. As previously mentioned in Section 1 of the current review, the mechanisms responsible for hepatic I/R and consequently the strategies required to regulate hepatic I/R damage might differ according to the duration of ischemia. Thus, the ischemic timing regime might be crucial in hepatic I/R studies evaluating the effects of, and potential relationship between (HSF1)-β-catenin axis and NLRP3 activity.

Treatment with 3,3’,5-triiodothyronine (T3) in livers submitted to 60 min of partial (70%) warm ischemia reduced the levels of NLRP3 and IL-1β [120]. This pharmacological strategy induced the expression of antioxidant (NF-κB, Nrf2), anti-apoptotic and acute-phase (NF-κB, STAT3), and cell proliferation (AP-1, STAT3) proteins [120,121,122,123,124]. The mitigation of I/R liver injury, including the anti-inflammatory response, using T3 was negated by the parallel administration of an AMPK inhibitor (compound C), resulting in aggravation of the hepatic damage. This study did not investigate the role of inflammasome in the changes induced. In addition, it should be considered that neither T3 nor the AMPK inhibitors used by the authors are drugs specifically designed to regulate inflammasome action. Another experimental strategy involved administering of docosahexaenoic acid, a main component of Omega (ω)-3 polyunsaturated fatty acids, which protects against I/R damage in livers undergoing 30 min of total warm I/R injury [125,126]. Evaluating the contribution to inflammasome regulation, the authors suggest that the underlying mechanisms of docosahexaenoic acid include activation of PI3K/Akt pathway and the inhibition of proteins such as NLRP3, ASC and cleaved caspase-1 involved in pyroptosis, a highly inflammatory form of non-apoptotic and caspase-1-dependent programmed cell death [126,127]. In addition, scrutiny of TUNEL staining suggested that docosahexaenoic acid suppressed pyroptosis [126]. However, it should be noted that DNA damage, marked by TUNEL staining, is identical in both pyroptosis and apoptosis [89]. Since pyroptosis is defined by the presence of both active caspase-1 and propidium iodide positivity [89], measurement of the aforementioned parameters by flow cytometry could be of interest to further characterize the presence and relevance of pyroptotic cell death in liver I/R injury in such conditions. Moreover, specific inhibitors of inflammasome need to be examined under conditions of different warm ischemia times and percentages. A recent study shows that treatment with Serp-2, a virus-derived inhibitor of apoptosis and inflammasome, regulates the levels of caspase-1, 8 and 10, improving the survival of mice submitted to 90 min of partial (70%) warm ischemia [128]. Interestingly, a study by Yang et al., using an experimental model of hepatic I/R based on 45 min of 70% warm ischemia, showed that Z-VD-fmk, a pan-caspase inhibitor (including caspase-1), had no effect on I/R injury or on the number of TUNEL-positive cells and staining pattern (nucleus and cytosol) [129]. The authors noted the minor role of apoptosis, thus contesting a relevant role for inflammasome or pyroptosis in hepatic I/R, at least in conditions of 90 min of partial (70%) warm ischemia [128] or 30 min of total warm I/R injury [126]. Data suggest that future research should be focused on detailing the type of cell death (necrosis, apoptosis and/or pyroptosis) and the signaling mechanisms of cell death, to identify specific targets for attenuating hepatic I/R injury. The administration of the Chinese herbal-derived Xuebijing in mice submitted to 90 min of partial (70%) ischemia did not induce changes in the expression of NLRP3 and ASC per se, but decreased the cleavage of pro-caspase-1 and pro-IL-1β, which is known to be the key step in the processing of mature IL-1β within the inflammasome [130]. Authors suggest that Xuebijing affected the assembly or function of inflammasome rather than the protein expression of inflammasome components and this ameliorated hepatic I/R injury [130]. However, this experiment offered no specific scrutiny of inflammasome action, and it should be noted that the hepatoprotective effect of Xuebijing could be partly due to inhibition of the expression of pro-inflammatory cytokines.

It has been well-documented that inflammatory immune responses diminish under conditions of food deprivation [131]. After 12 h of fasting, mice submitted to 60 min of partial (70%) warm ischemia showed reduced inflammatory responses, evidenced by increases in β-hydroxybutyric acid expression, up-regulation of acetylated histone-3 and the activation of Forkhead box protein O1 and HO-1. In addition, HMGB1 expression was reduced and NF-κB and NLRP3 inactivated [132]. Moreover, authors suggest that the up-regulation of autophagy induced by Forkhead box protein O1 may also play an important role in suppressing liver I/R injury. Starvation for 48–72 h reduced liver I/R injury by up-regulating anti-oxidative enzymes or autophagy and fasting for 1 day can prevent mouse liver I/R injury via the Sirtuin1-mediated down-regulation of circulating HMGB1 [133,134,135]. These findings strongly suggest the importance of dietary control for preventing I/R injury. However, fasting effects on hepatic I/R damage have yet to be interrogated for the specific role of inflammasome.

**Figure 2:**
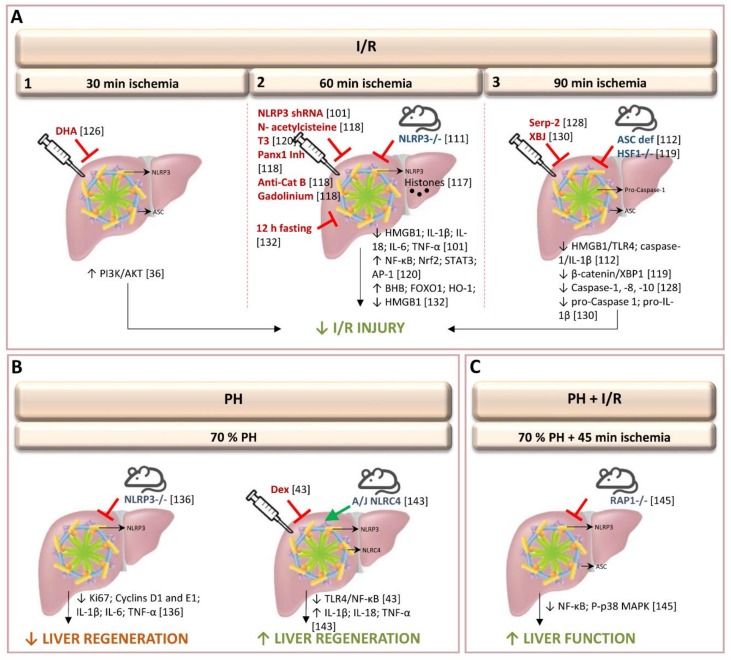
Schematic representation of the role of inflammasome in different experimental models of warm I/R injury. (**A**) Experimental models of 30 (A.1), 60 (A.2) and 90 (A.3) minutes of warm ischemia-reperfusion injury without hepatic resection. (**B**) Experimental models of partial hepatectomy without ischemia-reperfusion. (**C**) Experimental models of partial hepatectomy with 45 min of warm ischemia-reperfusion injury. Abbreviations: AKT, Protein kinase B; AP-1, Activator protein 1; ASC, Apoptosis-associated speck-like protein containing a CARD; BHB, β-hydroxybutyric acid; Cat B, Cathepsin B; Dex, Dexmedetomidine; DHA, Docosahexaenoic acid; FOXO1, Forkhead box protein O1; HMGB1, High mobility group box 1; HO-1, Heme oxygenase 1; HSF1, Heat shock transcription factor 1; IL, Interleukin; NF-κB, Nuclear factor kappa-light-chain-enhancer of activated B cells; NLRC4, NLR family CARD domain-containing protein 4; NLRP3, NLR pyrin domain containing protein 3; Nrf2, Nuclear factor erythroid 2-related factor 2; Panx1 Inh, Pannexin-1 inhibitor; RAP1, Repressor activator protein 1; STAT3, Signal transducer and activator of transcription 3; T3, 3,3’,5-triiodothyronine; TLR4, Toll-like receptor 4; TNF, Tumor necrosis factor; XBJ, Xuebijing; XBP1, X-box-binding protein 1.

The observations recorded above, summarized in Figure 2, emanate from studies of non-steatotic livers only. In our view, it is crucial to widen the parameters of research to incorporate parallel findings in relation to the presence of steatosis. Given that the prevalence of obesity ranges from 24–45% of the population, we expect a progressive increase in the appearance of steatosis in hepatic surgical practice. The use of experimental models that simulate as far as possible the surgical conditions present in clinical practice, including the use of steatotic livers, might help to identify the best therapeutic strategies to protect these types of liver. It is well known that steatotic livers exhibit greater regenerative failure response and reduced tolerance to I/R injury. The mechanisms involved in the pathology of I/R and consequently the therapeutic strategies that should be applied in the clinical surgery may vary with the presence or absence of steatosis.

#### 3.1.2. Role of Inflammasome in Experimental Models of Hepatectomy without Ischemia-Reperfusion

Activation of the inflammasome in the liver has been described after 70% partial hepatectomy (PH) [136]. Interestingly, after PH, the liver-to-body weight ratio and the expression of regenerative mediators (Ki67, and cyclins D1 and E1) and pro-inflammatory cytokines (IL-1β, TNF-α, and IL-6), showed a decrease in NLRP3-KO mice when compared to control mice [136]. The authors indicate that NLRP3 signaling is required for the induction of inflammatory response and the development of liver regeneration, after 70% PH [136]. Previous studies demonstrated that the adequate inflammatory response for optimal liver regeneration requires induction by inflammatory cytokines (IL-1β, TNF-α, and IL-6). However, liver regeneration is, itself, induced by TNF-α and IL-6, thus, the hypothesis that increased TNF-α and IL-6 contribute to NLRP3-mediated liver regeneration should be tested [137,138,139]. Interestingly, contradictory results have been described after dexmedetomidine treatment. Results in mice with 70% PH indicate that treatment with dexmedetomidine, a highly selective agonist of α2-adrenergic receptors, protected the liver against I/R injury via the suppression of the TLR4/NF-κB pathway. This promoted liver regeneration and liver function recovery via NLRP3 inflammasome inhibition [140]. It is well known that inflammation is usually beneficial for the host and leads to both neutralization of the causative factor and tissue recovery. However, under certain conditions, when “inflammatory machinery” is not properly orchestrated, inflammation may lead to significant pathology. The pathogenic role of inflammasome in many types of liver disease is well documented [141,142]. For instance, the NLRP3 inflammasomes, activated by telomere-independent repressor activator protein 1 (RAP1)/keratinocyte chemoattractant axis, played a critical role in initiating inflammation during the early stage of liver graft injury. Therefore, whether NLRP3 increased inflammatory response due to inflammasome activation is beneficial for liver recovery requires further investigation, especially, considering that the two studies mentioned above employed different strategies. Ando et al. [136] used a specific subject sample: NLRP3-KO mice, whereas Lv et al. [140] used an exogenous and non-specific treatment: dexmedetomidine. In addition, an experimental model of 70% PH in mice with genetically inbred resistance to CCl4-induced fibrosis (through A/J allele of Nlrc4, which modulates the resolution of hepatic fibrosis), found that the NLR family NLRC4 inflammasome-driven production of inflammatory cytokine signaling (TNF-α, IL-1β, and IL-18) led to benefits on damage and hepatocyte proliferation [143]. Further investigations characterizing the NLRC4 inflammasome and its association to liver regeneration will potentially provide new insights for treatment of liver disease. In addition, comparative studies evaluating the relevance of NLRP3 or NLRC4 regulators on liver regeneration after 70% PH should be evaluated.

Overall, results pertaining to the beneficial/detrimental effects of inflammasome activation on liver regeneration in 70% PH, are contradictory (Figure 2). Before insights for improving liver surgery can be properly interpreted, further studies are needed to isolate and compare the different strategies deployed and the effects of non-specific drugs, such as dexmedetomidine, in different subject samples and experimental models. Moreover, as with investigations in warm I/R, the studies mentioned above have been mainly focused in non-steatotic livers. As previously expounded, hepatic steatosis must be subjected to dedicated research if we are to gain the most far-reaching benefits for clinical practice.

#### 3.1.3. Role of Inflammasome in Experimental Models of Hepatic Resection under Vascular Occlusion

In previous sections: Section 3.1.1 and Section 3.1.2, we show that inflammasome inhibition could be beneficial in conditions of warm I/R, but inflammasome activation may be necessary for liver regeneration after PH. Of note, in clinical practice PH is usually performed under vascular occlusion (I/R) to control bleeding during parenchymal dissection [41]. In addition, the following should be noted: the existence of liver regeneration may alter the mechanisms of liver I/R injury, and hepatic I/R negatively affects liver regeneration. Thus, it may not be appropriate to extrapolate from the strategies reported to date aimed at regulating inflammasome activation in warm ischemia or PH, to infer comparable beneficial effects in conditions requiring PH under I/R. Experimental models reflecting common clinical conditions are needed. The ensuing development of damage-limiting therapeutic strategies could be implemented in clinical practice in relatively short order [144].

To our knowledge, only Liu et al. have addressed the role of the inflammasome in an experimental model of PH with I/R (Figure 2) [145]. Authors used wild-type and RAP1−/− mice subjected to partial hepatic I/R for 45 min and major PH [145,146]. In RAP1−/− mice, the expression levels of NLRP3, ASC, NF-κB, and P-p38 MAPK were down-regulated, in contrast to those observed in wild type. In addition, liver function improved and inflammatory response decreased in RAP1−/− mice [145]. Strategies targeting RAP1/NLRP3 inflammasome will need further experimentation to determine whether the changes induced potentially have clinical relevance for liver resections with I/R.

#### 3.1.4. Clinical Results of Inflammasome Activation in Liver Resection

A clinical study proposed inflammasome components as prognostic biomarkers after liver resection in HCC [147], which is both necessary and relevant in clinical practice. Expression of inflammasome components in non-tumorous tissue was evaluated for its influence on postsurgical HCC prognosis. Both primary HCC tumor tissue and surrounding corresponding non-tumorous tissues were analyzed. Interestingly, the expression of NLRP3, NLRC4, and AIM2 increased in corresponding normal tissue compared to that in HCC. Moreover, these results were significantly correlated with worse overall survival. The authors concluded that high expression of NLRP3, NLRC4, and caspase-1 in background non-tumorous liver significantly correlate with poor prognosis of patients after resection of HCC [147]. In addition, they found that higher expression of inflammasome component genes was related, not only to the pre-operative liver pathological status of the liver, such as decreased prothrombin time and viral infection type, but also to tumor factors including tumor size and growth type [147]. However, the study had limitations, not least, by virtue of being a single-institute retrospective. Further research into the underlying mechanisms involved in the effect of inflammasome on carcinogenesis or tumor malignancy is needed and could provide targets for HCC therapy.

### 3.2. Inflammasome in Cold Ischemia-Reperfusion Associated with Liver Transplantation

#### 3.2.1. Role of Inflammasome in Experimental Models of Ex Vivo Liver Transplantation

**Figure 3:**
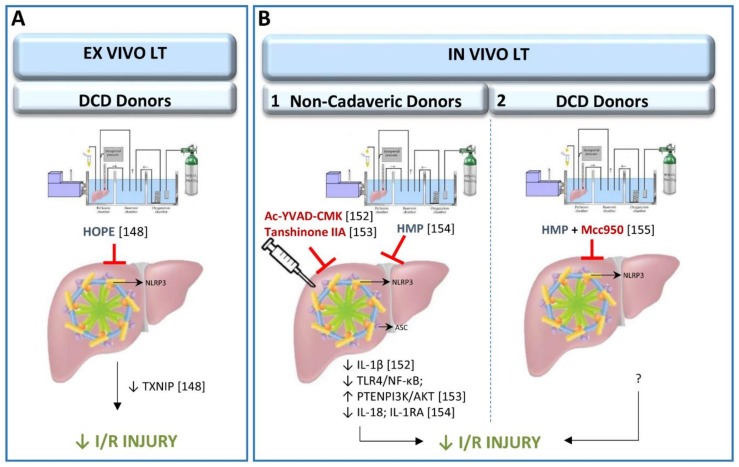
Schematic representation of the role of inflammasome in different experimental models of cold I/R injury. (**A**) ex vivo liver transplantation model. (**B**) in vivo liver transplantation from non-cadaverid donors (B.1) and from cardiac-circulatory death donors (B.2) models. Abbreviations: AKT, Protein kinase B; ASC, Apoptosis-associated speck-like protein containing a CARD; DCD, Cardiac-circulatory death; HOPE, Hypothermic oxygenated perfusion; HMP, Hypothermic machine perfusion; IL, Interleukin; IL-1RA, IL-1R antagonist; LT, liver transplantation; NF-κB, Nuclear factor kappa-light-chain-enhancer of activated B cells; NLRP3, NLR pyrin domain containing protein 3; PI3K, Phosphoinositide 3-kinase; PTEN, Phosphatase and tensin homolog; TXNIP, Thioredoxin-interacting protein; TLR4, Toll-like receptor 4.

The NLRP3 inflammasome pathway has recently been studied in cold storage conditions or hypothermic oxygenated perfusion (HOPE) machine in an ex vivo LT model from cardiac-circulatory death (DCD) donors [148] (Figure 3). HOPE is a relatively new dynamic preservation procedure which improves liver grafts through different mechanisms, one of which may be the inhibition of the thioredoxin-interacting protein/NLRP3 inflammasome pathway [149,150,151]. In this ex vivo experimental study, rat livers were submitted to 30 min of warm ischemia, followed by 3 h with HOPE and only up to 1 h of isolated reperfusion [148]. It is important to mention that for the evaluation of drugs with potential therapeutic effects in LT, the use of an in vivo LT model is recognized as the standard method. Indeed, the lack of blood in the ex vivo experimental model cannot simulate the surgical conditions occurring in clinical LT. Notwithstanding, the use of HOPE could be replicated in an in vivo experimental model of LT, to induce NLRP3 inflammasome activation regulation. In the following section we describe such an experiment, with a prolonged reperfusion period, as commonly used in clinical practice.

#### 3.2.2. Role of Inflammasome in Experimental Models of In Vivo Liver Transplantation

The up-regulation of ASC in grafts submitted to LT induces the production of IL-1β, which mediates the inflammatory response. In addition, treatment with Ac-YVAD-CMK, a selective irreversible inhibitor of caspase-1, reduced the production of IL-1β and attenuated inflammation in recipients [152]. Interestingly, Ac-YVAD-CMK has also reduced the expression of ASC, suggesting that some feedback mechanisms could be operating. This possibility should be further investigated since the derived results may provide new therapeutic targets in the clinical setting. Similarly, I/R injury was mitigated and inflammatory cytokines (TNF-α, IL-4, IL-10 and TGF-β) regulated, in an experimental model of LT using pre-treatment with tanshinone IIA, an active compound derived from traditional Chinese medicine [153]. These effects were attributed to down-regulation of the TLR-4/NF-κB/NLRP3 pathway, thus promoting activation of the PTEN/PI3K/AKT-PCNA axis in Kupffer cells [153]. It should be noted that these results could equally be the result of improvement in the liver graft quality prior to removal from the donor, as to improvement following implantation, because the pre-treatment with tanshinone IIA was administered to both donors and recipients one week before LT was performed. Thus, further investigations, aimed at differentiating the effects of tanshinone IIA when administered in either donors or recipients as well as in both donors and recipients, could be of scientific and clinical interest. Research focused on developing therapeutic strategies aimed at improving the quality of liver grafts before implantation in the recipient could yield a double benefit. Not only would survival rates and post-operative recovery improve, but the donor pool could be significantly broadened by upgrading the health status of the donor organ prior to removal. In large animal models, inflammasome activation has been studied in allografts preserved in machine perfusion and submitted to LT [154]. Results indicated that NLRP3 inflammasome regulates inflammatory response (reducing IL-18 and increasing IL-1RA levels) [154]. Experimental LT with large animals, to evaluate the role of NLRP3 inflammasome activity needs to be conducted by submitting liver grafts to static cold storage rather than machine perfusion. It is the most commonly used organ preservation method. Static cold storage involves flushing the procured organ with preservation solution at 0–4 °C, then immersing it into preservation solution at the same temperature until transplantation, whereas machine perfusion involves pulsatile perfusion of the liver with a hypothermic perfusate or with a normothermic perfusate.

Further investigations will be necessary to evaluate whether these data mentioned above, which have been evaluated in in vivo LT from non-cadaveric donors [152,153,154] might be also extrapolated in preclinical studies from cadaveric donors, in order to mimic the surgical clinical conditions. Indeed, the results obtained in non-cadaveric donors might be different to those obtained from cadaveric donors. Deceased donation comprises two types: donation after DCD and donation after brain death (BD); the fundamental distinction being the diagnostic criteria for death. DCD refers to death confirmed using circulatory criteria. The standard model for organ retrieval from deceased donation however is BD, which refers to death using neurological criteria. The potential contribution of DCD to overall deceased donor numbers varies internationally (4–20% of transplanted grafts). However, in clinical practice, around 80% of grafts are currently procured from brain-dead donors. Briefly, BD and cardiac arrest induce the release of various pro-inflammatory mediators, up-regulation of adhesion molecules on vascular endothelium and subsequent leukocyte tissue infiltration. This might affect the quality of liver grafts and the post-operative outcomes. Indeed, protective strategies, such as preconditioning, which are able to reduce damage in LT from non-BD donors is not useful in the presence of BD. This might also occur with NLRP3 inflammasome regulators.

In addition to the studies of LT from non-cadaveric donors mentioned above (summarized in Figure 3), the NLRP3 inflammasome activation has been studied in a porcine LT from DCD donors. Organs were submitted to 30 min of warm ischemia, 2 h of cold ischemia and preserved in a hypothermic machine perfusion system for 2 h before the implantation of liver grafts in the recipient. Mcc950, a novel NLRP3-inflammasome selective small-molecule inhibitor, was added to the perfusate of the hypothermic machine perfusion and administered to recipients [155]. The study shows that the addition of Mcc950 improved the outcomes of LT from DCD donors via inhibition of the inflammatory response, reduction of hepatocyte apoptosis, and improvement of liver function (Figure 3) [155]. In the context of clinical practice, the encouraging results of this experiment in regulating NLRP3 activity in DCD donations with short ischemic periods, urges increased efforts to minimize both warm ischemia and cold ischemia times during DCD organ procurement and transplantation in order to improve the viability of liver grafts and recipients after LT [156,157]. Further investigation of the role of the NLRP3 inflammasome pathway in LT from DCD donors is recommended to assess the potential for replicating these results in LT from BD donors. However, major differences between DCD and BD have been observed regarding the mechanisms that procure detrimental effects in LT post-operative outcomes [158].

#### 3.2.3. Clinical Results of Inflammasome Activation in Liver Transplantation

In a review of 76 liver transplant patients, Liu et al. examined standard and fatty liver grafts from living donors after LT [145]. Interestingly, the intragraft expression of both NLRP3 inflammasome and telomere-independent RAP1, were up-regulated post-transplantation in fatty liver grafts from living donors. Moreover, overexpression of RAP1/NLRP3 was strongly associated with poor liver function characterized by high levels of transaminases and urea, as well as neutrophil infiltration after LT. It is well known that severe steatosis is associated with a higher incidence of graft failure after LT [159]. The increased susceptibility of fatty livers to I/R injury and poor post-LT functioning might, these authors suggest, depend on this up-regulation in RAP1/NLRP3 expression [145]. They further propose, following a study indicating that fatty acids activated NLRP3 inflammasomes to stimulate the immune cells in mice [91], that NLRP3 inflammasome is responsible for the susceptibility of steatotic liver grafts to I/R injury. However, further preclinical and clinical investigations will be necessary to confirm this, since in the study did not examine the effects of regulation of NLRP3 on hepatic damage in steatotic grafts.

A clinical study of 190 patients, which focused on post-transplantation bacterial infection identified donor gene polymorphisms involved in NLRP3 inflammasome activation as representing a risk of bacterial infection prior to surgery [160]. In fact, polymorphisms in C7 (for soluble membrane attack complex formation) and mannan-binding lectin, were associated with bacterial infection and with decreased levels of recipient C7 protein expression, soluble membrane attack complex, and IL-1β. Anti-bacterial defense mechanisms of C7 may, the authors propose, involve membrane attack complex formation, since in in vitro experiments have shown that the membrane attack complex triggered NLRP3 inflammasome activation and IL-1β release [160]. However, as suggested by the authors, these results require further evaluation in other larger cohort studies.

The indication that up-regulated NLRP3 inflammasome is implicated in the increased vulnerability of living donor steatotic livers to I/R injury and bacterial infection in LT is reflected in clinical hepatic resection practice. Furthermore, the high expression of hepatic NLRP3 correlates with poor prognosis of patients after resection of HCC.

## 4. Future Perspectives and Conclusion

I/R injury and impaired regeneration associated with resection and LT, especially in steatotic livers, has been for many years an unsolved problem in clinical practice. Dedicated research needs to focus on the role of inflammasome and potential regulatory regimes to improve liver surgery outcomes. Special attention should be paid to the different types of livers (steatotic and non-steatotic) and the different surgical procedures (PH with I/R and LT from BD, DCD or living donors). A better understanding will help increase the quality and viability of organs submitted to surgery, reduce post-operative problems, and increase the availability of suitable grafts for transplantation, which will ultimately reduce the waiting lists.

Controversial results have been described in experimental models of warm I/R and PH. Thus, it has been reported that NLRP3 contributes to hepatic I/R injury independently of the ASC, whereas other studies describe ASC activation after warm I/R. These contradictory results could be explained by the use of different durations of I/R (60 or 90 min). In addition, whereas several authors indicate that the inhibition of inflammasome activation in conditions of warm I/R reduces caspase-1/IL-1β, TNF, IL-18 and HMGB1 production and, this in turn protects against hepatic damage, whether through NLRP3 or ASC, other authors indicate that inflammasome activation could be necessary for liver regeneration after PH. Interestingly, in clinical practice, PH is usually performed under vascular occlusion. The only experimental study focused in PH with I/R suggests targeting RAP1/NLRP3 inflammasomes to protect the liver against I/R injury. What is clear is that results from both clinical practice and experimental studies to date have provided much new ground for investigation into the role of the inflammasome in liver resections. In our view, appropriate experimental models of liver resections and the use of specific drugs, without side effects, aimed at regulating inflammasome activation in such surgical procedures might be of scientific and clinical interest.

Preclinical results in LT from non-steatotic livers and without the presence of BD, suggest that inhibition of ASC or NLRP3 inflammasome pathways may attenuate liver I/R injury. However, in clinical practice, BD represents the main source of LT donation. BD is a detrimental condition for organ drafts and its effects may be responsible for disruptive change in the inflammasome pathway. Thus, further studies will be required to identify whether reported research results might be mirrored in LT from BD donors.

Clinical data suggest that NLRP3, NLRC4, and caspase-1 expression correlates with poor prognosis in patients after resection of HCC. However, there has been, to date, very little analysis or scrutiny of clinical PH and LT practice for indications of the role of inflammasome in different outcomes. In our view, future research in similar experimental models that closely reproduce the clinical conditions (i.e., experimental models of HCC and hepatectomy under vascular occlusion) will be required to understand the involvement of the inflammasome in the pathophysiology of I/R in liver resections. Such research, and the consequential development of protective interventions, would enable the more successful transposition of therapeutic strategies designed in animal models to the hospital ward and operating theatre. Likewise, in LT from living donors, the up-regulation of NLRP3 inflammasome is considered as a risk factor for poor tolerance of I/R injury in steatotic livers. Further investigations will also be required to elucidate whether the changes in NLRP3 inflammasome observed in steatotic LT from living donors are also evidenced in LT from DCD or BD donors. Considering that progress in the study of human subjects is slow, the majority of human tissues are not being routinely accessible for research, the use of experimental models is the best options for examining the relevance of inflammasome in hepatic I/R injury. We recognize the very real complications of transferring research knowledge between applications i.e., into clinical practice. Multidisciplinary research groups could usefully devote additional efforts to evaluating the role of inflammasome in experimental models of LT that simulate as closely as possible the real clinical conditions (e.g., using grafts from DCD or BD donors, with properly calibrated ischemia times, among other factors) to better understand the pathophysiology of hepatic I/R, especially in steatotic liver grafts. The ultimately aim must be to develop therapeutic strategies aimed at improving graft viability, thus increasing the organ donor pool and the post-operative outcomes after LT.

In hepatic I/R associated with hepatic resections and LT, and in line with suggestions made by other authors in different pathologies [69], future research should be focused on elucidating the molecular mechanisms of inflammasome activation in order to identify specific and effective NLRP3 inhibitors or inhibitory pathways and assess their therapeutic potential. A number of NLRP3 inhibitors have been reported to date, including those that either directly inhibit NLRP3 or indirectly inhibit inflammasome components or related signaling events.

It has been reported that the high expression of inflammasome components in non-tumorous liver tissue of HCC might provide good prognostic biomarkers in curatively HCC resection. In the clinical practice of hepatic resections and LT, standard therapeutic approaches do not reference changes to inflammasome components or prognostic biomarkers such as NLRP3 or caspase-1. Addressing this issue in future investigations might be of clinical and scientific relevance. The clinical field suffers currently from a paucity of reliable, accessible markers for predicting the risks of post-operative failure, loss of function and infection, among others in, for example, the transplantation of livers with steatosis. Indeed, transaminases and specifically alanine aminotransferase, are commonly used as liver damage markers, despite compromised reliability given that some patients suffering from non-alcoholic fatty liver disease do not present high alanine aminotransferase levels [161]. During procurement of the graft in the donor, the gold standard to assess hepatic steatosis is a histological analysis [35]. Nevertheless, it is important to note that liver biopsy has a significant interobserver variability and is inadequate for continuous monitoring. The use of sensitive and specific biomarkers could simplify liver injury assessment and improve the timely management of post-operative complications, especially in surgery involving steatotic livers.

Additional investigations are required to elucidate whether PAMPs derived from the gut and DAMPS derived from the liver during the splanchnic congestion that occurs in the anhepatic phase of LT might negatively affect the viability of liver grafts submitted to LT. If this is the case, the regulation of inflammasome activation might be useful to reduce both local and systemic inflammatory responses associated with LT. Determination of the mechanisms involved and the feasibility and value of the different regulatory procedures available should be directly applicable to clinical practice in liver resections and LT.

## Abbreviations

AIM2	Absent in melanoma 2
AKT	Protein kinase B
AP-1	Activator protein 1
ASC	Apoptosis-associated speck-like protein containing a caspase recruitment domain
ATP	Adenosine triphosphate
BD	Brain death
BHB	β-hydroxybutyric acid
CARD	Carboxy-terminal caspase recruitment domain
Cat B	Cathepsin B
CCl4	Carbon tetrachloride
DAMPs	Damage-associated molecular patterns
DCD	Cardiac-circulatory death
Dex	Dexmedetomidine
DHA	Docosahexaenoic acid
FOXO1	Forkhead box protein O1
HCC	Hepatocellular carcinoma
HMGB1	High mobility group box 1
HMP	Hypothermic machine perfusion
HO-1	Heme oxygenase 1
HOPE	Hypothermic oxygenated perfusion
HSF1	Heat shock transcription factor 1
I/R	Ischemia-reperfusion
IL	Interleukin
IL-1R	Interleukin-1 receptor
IL-1RA	IL-1R antagonist
LT	Liver transplantation
NF-κB	Nuclear factor kappa-light-chain-enhancer of activated B cells
NLR	NOD-like receptors
NLRC4	NLR family CARD domain-containing protein 4
NLRP	NLR pyrin domain containing protein
NOD	Nucleotide-binding oligomerization domain
Nrf2	Nuclear factor erythroid 2-related factor 2
PAMPs	Pathogen-associated molecular patterns
Panx1 Inh	Pannexin-1 inhibitor
PH	Partial hepatectomy
PI3K	Phosphoinositide 3-kinase
PTEN	Phosphatase and tensin homolog
RAP1	Repressor activator protein 1
ROS	Reactive oxygen species
STAT3	Signal transducer and activator of transcription 3
T3	3,3’,5-triiodothyronine
TGF-β	Transforming growth factor-β
TLR	Toll-like receptor
TNF	Tumor necrosis factor
TXNIP	Thioredoxin-interacting protein
XBJ	Xuebijing
XBP1	X-box-binding protein 1

## References

[1] Peralta C., Jiménez-Castro M.B., Gracia-Sancho J. (2013). Hepatic ischemia and reperfusion injury: Effects on the liver sinusoidal milieu. J. Hepatol..

[2] Fu P., Li W., Muriel P. (2017). Nitric oxide in liver ischemia-reperfusion injury. Liver Pathophysiology.

[3] Selzner N., Rudiger H., Graf R., Clavien P. (2003). Protective strategies against ischemic injury of the liver. Gastroenterology.

[4] Jaeschke H. (2003). Molecular mechanisms of hepatic ischemia-reperfusion injury and preconditioning. Am. J. Physiol. Gastrointest Liver Physiol..

[5] Montalvo-Jave E.E., Escalante-Tattersfield T., Ortega-Salgado J.A., Piña E., Geller D.A. (2008). Factors in the pathophysiology of the liver ischemia-reperfusion injury. J. Surg. Res..

[6] Gracia-Sancho J., Villarreal Jr G., Zhang Y., Yu J.X., Liu Y., Tullius S.G., García-Cardeña G. (2010). Flow cessation triggers endothelial dysfunction during organ cold storage conditions: Strategies for pharmacologic intervention. Transplantation.

[7] Gracia-Sancho J., Casillas-Ramírez A., Peralta C. (2015). Molecular pathways in protecting the liver from ischaemia/reperfusion injury: A 2015 update. Clin. Sci..

[8] Papadopoulos D., Siempis T., Theodorakou E., Tsoulfas G. (2013). Hepatic ischemia and reperfusion injury and trauma: Current concepts. Arch. Trauma. Res..

[9] Bilzer M., Gerbes A.L. (2000). Preservation injury of the liver: Mechanisms and novel therapeutic strategies. J. Hepatol..

[10] Mckeown C.M., Edwards V., Phillips M.J., Harvey P.R., Petrunka C.N., Strasberg S.M. (1988). Sinusoidal lining cell damage: The critical injury in cold preservation ofliver allografts in the rat. Transplantation.

[11] Ikeda T., Yanaga K., Kishikawa K., Kakizoe S., Shimada M., Sugimachi K. (1992). Ischemic injury in liver transplantation: Difference in injury sites between warm and cold ischemia in rats. Hepatology.

[12] Peralta C., Bartrons R., Riera L., Manzano A., Xaus C., Gelpí E., Rosello-Catafau J. (2000). Hepatic preconditioning preserves energy metabolism during sustained ischemia. Am. J. Physiol. Gastrointest Liver Physiol..

[13] Gasbarrini A., Borle A.B., Farghali H., Bender C., Francavilla A., Van Thiel D. (1992). Effect of anoxia on intracellular ATP, Na+i, Ca2+i, Mg2+i, and cytotoxicity in rat hepatocytes. J Biol. Chem..

[14] Caraceni P., Domenicali M., Vendemiale G., Grattagliano I., Pertosa A., Nardo B., Moselli-Labate A.M., Trevisani F., Palasciano G., Altomare E. (2005). The reduced tolerance of rat fatty liver to ischemia reperfusion is associated with mitochondrial oxidative injury. J. Surg. Res..

[15] Caldwell-Kenkel J.C., Currin R.T., Tanaka Y., Thurman R.G., Lemasters J.J. (1989). Reperfusion injury to endothelial cells following cold ischemic storage of rat livers. Hepatology.

[16] Huet P.M., Nagaoka M.R., Desbiens G., Tarrab E., Brault A., Bralet M.P., Bilodeau M. (2004). Sinusoidal endothelial cell and hepatocyte death following cold ischemia-warm reperfusion of the rat liver. Hepatology.

[17] Marzi I., Zhong Z., Lemasters J.J., Thurman R.G. (1989). Evidence that graft survival is not related to parenchymal cell viability in rat liver transplantation. The importance of nonparenchymal cells. Transplantation.

[18] Kukan M., Haddad P.S. (2001). Role of hepatocytes and bile duct cells in preservation-reperfusion injury of liver grafts. Liver Transpl..

[19] Ramalho F., Alfany-Fernandez I., Casillas-Ramírez A., Massip-Salcedo M., Serafín A., Rimola A., Arroyo V., Rodes J., Rosello-Catafau J., Peralta C. (2009). Are angiotensin II receptor antagonists useful strategies in steatotic and nonsteatotic livers in conditions of partial hepatectomy under ischemia-reperfusion?. J. Pharmacol. Exp. Ther..

[20] Hayashi H., Chaudry I., Clemens M., Baue A. (1986). Hepatic ischemia models for determining the effects of ATP-MgCl2 treatment. J. Surg. Res..

[21] Cannistrà M., Ruggiero M., Zullo A., Gallelli G., Serafini S., Maria M., Naso A., Grande R., Serra R., Nardo B. (2016). Hepatic ischemia reperfusion injury: A systematic review of literature and the role of current drugs and biomarkers. Int. J. Surg..

[22] Chaudry I., Clemens M., Ohkawa M., Schleck S., Baue A.E. (1982). Restoration of hepatocellular function and blood flow following hepatic ischemia with ATP–MgCl2. Adv. Shock Res..

[23] Hasselgren P., Jennische E., Fornander J., Hellman A. (1982). No beneficial affect of ATPMgCl2 on impaired transmembrane potential and protein synthesis in liver ischemia. Acta Chir. Scand..

[24] Gonzalez-Flecha B., Cutrin J., Boveris A. (1993). Time course and mechanism of oxidative stress and tissue damage in rat liver subjected to in vivo ischemia-reperfusion. J. Clin. Invest..

[25] Kawachi S., Hines I.N., Laroux F.S., Hoffman J., Bharwani S., Gray L., Leffer D., Grisham M.B. (2000). Nitric oxide synthase and postischemic liver injury. Biochem. Biophys. Res. Commun..

[26] Kuboki S., Shin T., Huber N., Eismann T., Galloway E., Schuster R., Blanchard J., Edwards M.J., Lentsch A.B. (2008). Hepatocyte signaling through CXC chemokine receptor-2 is detrimental to liver recovery after ischemia/reperfusion in mice. Hepatology.

[27] van Riel W.G., van Golen R.F., Reiniers M.J., Heger M., van Gulik T.M. (2016). How much ischemia can the liver tolerate during resection?. Hepatobiliary Surg. Nutr..

[28] Olthof P.B., van Golen R.F., Meijer B., van Beek A.A., Bennink R.J., Verheij J., van Gulik T.M., Heger M. (2017). Warm ischemia time-dependent variation in liver damage, inflammation, and function in hepatic ischemia/reperfusion injury. Biochim. Biophys. Acta Mol. Basis. Dis..

[29] Fernández L., Heredia N., Grande L., Gómez G., Rimola A., Marco A., Gelpí E., Roselló-Catafau J., Peralta C. (2002). Preconditioning protects liver and lung damage in rat liver transplantation: Role of xanthine/xanthine oxidase. Hepatology.

[30] D’Alessandro A.M., Kalayoglu M., Sollinger H.W., Hoffmann R.M., Reed A., Knechtle S.J., Pirsch J.D., Hafez G.R., Lorentzen D., Belzer F.O. (1991). The predictive value of donor liver biopsies for the development of primary nonfunction after orthotopic liver transplantation. Transplantation.

[31] Loinaz C., Gonzalez E.M. (2000). Marginal donors in liver transplantation. Hepatogastroenterology.

[32] Rinella M.E., Alonso E., Rao S., Whitington P., Fryer J., Abecassis M., Superina R., Flamm S.L., Blei A.T. (2001). Body mass index as a predictor of hepatic steatosis in living liver donors. Liver Transpl..

[33] Busuttil R.W., Tanaka K. (2003). The utility of marginal donors in liver transplantation. Liver Transpl..

[34] Crowley H., Lewis W.D., Gordon F., Jenkins R., Khettry U. (2000). Steatosis in donor and transplant liver biopsies. Hum. Pathol..

[35] McCormack L., Dutkowski P., El-Badry A.M., Clavien P.A. (2011). Liver transplantation using fatty livers: Always feasible?. J. Hepatol..

[36] Wong T.C., Fung J.Y., Chok K.S., Cheung T.T., Chan A.C., Sharr W.W., Dai W.C., Chan S.C., Lo C.M. (2016). Excellent outcomes of liver transplantation using severely steatotic grafts from brain-dead donors. Liver Transpl..

[37] Patel Y.A., Berg C.L., Moylan C.A. (2016). Nonalcoholic fatty liver disease: Key considerations before and after liver transplantation. Dig. Dis. Sci..

[38] Canelo R., Braun F., Sattler B., Klinge B., Lorf T., Ramadori G., Ringe B. (1999). Is a fatty liver dangerous for transplantation?. Transplant Proc..

[39] Tashiro H., Kuroda S., Mikuriya Y., Ohdan H. (2014). Ischemia-reperfusion injury in patients with fatty liver and the clinical impact of steatotic liver on hepatic surgery. Surg. Today.

[40] Spitzer A.L., Lao O.B., Dick A.A., Bakthavatsalam R., Halldorson J.B., Yeh M.M., Upton M.P., Reyes J.D., Perkins J.D. (2010). The biopsied donor liver: Incorporating macrosteatosis into high-risk donor assessment. Liver Transpl..

[41] McCormack L., Petrowsky H., Jochum W., Furrer K., Clavien P.A. (2007). Hepatic steatosis is a risk factor for postoperative complications after major hepatectomy: A matched case-control study. Ann. Surg..

[42] Veteläinen R., van Vliet A., Gouma D.J., van Gulik T.M. (2007). Steatosis as a risk factor in liver surgery. Ann. Surg..

[43] Clavien P.A., Yadav S., Sindram D., Bentley R.C. (2000). Protective effects of ischemic preconditioning for liver resection performed under inflow occlusion in humans. Ann. Surg..

[44] Behrns K.E., Tsiotos G.G., DeSouza N.F., Krishna M.K., Ludwig J., Nagorney D.M. (1998). Hepatic steatosis as a potential risk factor for major hepatic resection. J. Gastrointest Surg..

[45] de Meijer V.E., Kalish B.T., Puder M., Ijzermans J.N. (2010). Systematic review and meta-analysis of steatosis as a risk factor in major hepatic resection. Br. J. Surg..

[46] Bachellier P., Rosso E., Pessaux P., Oussoultzoglou E., Nobili C., Panaro F., Jaeck D. (2011). Risk factors for liver failure and mortality after hepatectomy associated with portal vein resection. Ann. Surg..

[47] Hamady Z.Z., Rees M., Welsh F.K., Toogood G.J., Prasad K.R., John T.K., Lodge J.P. (2013). Fatty liver disease as a predictor of local recurrence following resection of colorectal liver metastases. Br. J. Surg..

[48] Truant S., Bouras A.F., Petrovai G., Buob D., Ernst O., Boleslawski E., Hebbar M., Pruvot F.R. (2013). Volumetric gain of the liver after major hepatectomy in obese patients: A case-matched study in 84 patients. Ann. Surg..

[49] Ramos E., Torras J., Lladó L., Rafecas A., Serrano T., Lopez-Gordo S., Busquets J., Fabregat J. (2016). The influence of steatosis on the short- and long-term results of resection of liver metastases from colorectal carcinoma. HPB (Oxford).

[50] Jarnagin W.R., Gonen M., Fong Y., DeMatteo R.P., Ben-Porat L., Little S., Corvera C., Weber S., Blumgart L.H. (2002). Improvement in perioperative outcome after hepatic resection: Analysis of 1,803 consecutive cases over the past decade. Ann. Surg..

[51] Ijaz S., Yang W., Winslet M.C., Seifalian A.M. (2003). Impairment of hepatic microcirculation in fatty liver. Microcirculation.

[52] Peralta C., Brenner C. (2011). Endoplasmic reticulum stress inhibition enhances liver tolerance to ischemia/reperfusion. Curr. Med. Chem..

[53] Alfany-Fernandez I., Casillas-Ramirez A., Bintanel-Morcillo M., Brosnihan K.B., Ferrario C.M., Serafin A., Rimola A., Rodés J., Roselló-Catafau J., Peralta C. (2009). Therapeutic targets in liver transplantation: Angiotensin II in nonsteatotic grafts and angiotensin-(1-7) in steatotic grafts. Am. J. Transplant.

[54] Casillas-Ramírez A., Alfany-Fernández I., Massip-Salcedo M., Juan M.E., Planas J.M., Serafín A., Pallàs M., Rimola A., Rodés J., Peralta C. (2011). Retinol-binding protein 4 and peroxisome proliferator-activated receptor-γ in steatotic liver transplantation. J. Pharmacol. Exp. Ther..

[55] Jiménez-Castro M.B., Elias-Miro M., Mendes-Braz M., Lemoine A., Rimola A., Rodés J., Casillas-Ramírez A., Peralta C. (2012). Tauroursodeoxycholic acid affects PPARγ and TLR4 in Steatotic liver transplantation. Am. J. Transplant.

[56] Jiménez-Castro M.B., Casillas-Ramírez A., Mendes-Braz M., Massip-Salcedo M., Gracia-Sancho J., Elias-Miró M., Rodés J., Peralta C. (2013). Adiponectin and resistin protect steatotic livers undergoing transplantation. J. Hepatol..

[57] Fernández L., Carrasco-Chaumel E., Serafín A., Xaus C., Grande L., Rimola A., Roselló-Catafau J., Peralta C. (2004). Is ischemic preconditioning a useful strategy in steatotic liver transplantation?. Am. J. Transplant.

[58] Liu P., Xu B., Hock C.E. (2001). Inhibition of nitric oxide synthesis by L-name exacerbates acute lung injury induced by hepatic ischemia-reperfusion. Shock.

[59] Hato S., Urakami A., Yamano T., Uemura T., Ota T., Hirai R., Shimizu N. (2001). Attenuation of liver and lung injury after hepatic ischemia and reperfusion by a cytokine-suppressive agent, FR167653. Eur. Surg. Res..

[60] Wanner G.A., Ertel W., Muller P., Höfer Y., Leiderer R., Menger M.D., Messmer K. (1996). Liver ischemia and reperfusion induces a systemic inflammatory response through Kupffer cell activation. Shock.

[61] Watanabe M., Yamaguchi K., Chijiiwa K., Tanaka M. (2001). FR167653 improves survival and pulmonary injury after partial hepatectomy under ischemia/reperfusion in rats. J. Surg. Res..

[62] Franco-Gou R., Roselló-Catafau J., Peralta C. (2006). Protection against lung damage in reduced-size liver transplantation. Crit. Care Med..

[63] Cornide-Petronio M.E., Jiménez-Castro M.B., Gracia-Sancho J., Peralta C., Tsoulfas G. (2019). Ischemic preconditioning directly or remotely applied on the liver to reduce ischemia-reperfusion injury in resections and transplantation. Liver Disease and Surgery.

[64] Szabo G., Petrasek J. (2015). Inflammasome activation and function in liver disease. Nat. Rev. Gastroenterol. Hepatol..

[65] Bauernfeind F.G., Horvath G., Stutz A., Alnemri E.S., MacDonald K., Speert D., Fernandes-Alnemri T., Wu J., Monks B.G., Fitzgerald K.A. (2009). Cutting edge: NF-kappaB activating pattern recognition and cytokine receptors license NLRP3 inflammasome activation by regulating NLRP3 expression. J. Immunol..

[66] Franchi L., Eigenbrod T., Núñez G. (2009). Cutting edge: TNF-alpha mediates sensitization to ATP and silica via the NLRP3 inflammasome in the absence of microbial stimulation. J. Immunol..

[67] Xing Y., Yao X., Li H., Xue G., Guo Q., Yang G., An L., Zhang Y., Meng G. (2017). Cutting Edge: TRAF6 Mediates TLR/IL-1R Signaling-Induced Nontranscriptional Priming of the NLRP3 Inflammasome. J. Immunol..

[68] Tannahill G.M., Curtis A.M., Adamik J., Palsson-McDermott E.M., McGettrick A.F., Goel G., Frezza C., Bernard N.J., Kelly B., Foley N.H. (2013). Succinate is an inflammatory signal that induces IL-1β through HIF-1α. Nature.

[69] Swanson K.V., Deng M., Ting J.P. (2019). The NLRP3 inflammasome: Molecular activation and regulation to therapeutics. Nat. Rev. Immunol..

[70] Wree A., Marra F. (2016). The inflammasome in liver disease. J. Hepatol..

[71] Kubes P., Mehal W.Z. (2012). Sterile inflammation in the liver. Gastroenterology.

[72] Mohamadi Y., Mousavi M., Khanbabaei H., Salarinia R., Javankiani S., Hassanzadeh G., Momeni F. (2018). The role of inflammasome complex in ischemia-reperfusion injury. J. Cell Biochem..

[73] Schroder K., Tschopp J. (2010). The inflammasomes. Cell.

[74] Lamkanfi M. (2011). Emerging inflammasome effector mechanisms. Nat. Rev. Immunol..

[75] Sagulenko V., Thygesen S.J., Sester D.P., Idris A., Cridland J.A., Vajjhala P.R., Roberts T.L., Schroder K., Vince J.E., Hill J.M. (2013). AIM2 and NLRP3 inflammasomes activate both apoptotic and pyroptotic death pathways via ASC. Cell. Death Differ..

[76] Aachoui Y., Sagulenko V., Miao E.A., Stacey K.J. (2013). Inflammasome-mediated pyroptotic and apoptotic cell death, and defense against infection. Curr. Opin. Microbiol..

[77] Szabo G., Csak T. (2012). Inflammasomes in liver diseases. J. Hepatol..

[78] Muruve D.A., Pétrilli V., Zaiss A.K., White L.R., Clark S.A., Ross P.J., Parks R.J., Tschopp J. (2008). The inflammasome recognizes cytosolic microbial and host DNA and triggers an innate immune response. Nature.

[79] Hornung V., Ablasser A., Charrel-Dennis M., Bauernfeind F., Horvath G., Caffrey D.R., Latz E., Fitzgerald K.A. (2009). AIM2 recognizes cytosolic dsDNA and forms a caspase-1-activating inflammasome with ASC. Nature.

[80] Nakahira K., Haspel J.A., Rathinam V.A., Lee S.J., Dolinay T., Lam H.C., Englert J.A., Rabinovitch M., Cernadas M., Kim H.P. (2011). Autophagy proteins regulate innate immune responses by inhibiting the release of mitochondrial DNA mediated by the NALP3 inflammasome. Nat. Immunol..

[81] Rathinam V.A., Jiang Z., Waggoner S.N., Sharma S., Cole L.E., Waggoner L., Vanaja S.K., Monks B.G., Ganesan S., Latz E. (2010). The AIM2 inflammasome is essential for host defense against cytosolic bacteria and DNA viruses. Nat. Immunol..

[82] Poeck H., Bscheider M., Gross O., Finger K., Roth S., Rebsamen M., Hannesschläger N., Schlee M., Rothenfusser S., Barchet W. (2010). Recognition of RNA virus by RIG-I results in activation of CARD9 and inflammasome signaling for interleukin 1 beta production. Nat. Immunol..

[83] Miao E.A., Mao D.P., Yudkovsky N., Bonneau R., Lorang C.G., Warren S.E., Leaf I.A., Aderem A. (2010). Innate immune detection of the type III secretion apparatus through the NLRC4 inflammasome. Proc. Natl. Acad. Sci. USA.

[84] Mariathasan S., Newton K., Monack D.M., Vucic D., French D.M., Lee W.P., Roose-Girma M., Erickson S., Dixit V.M. (2004). Differential activation of the inflammasome by caspase-1 adaptors ASC and Ipaf. Nature.

[85] Vinzing M., Eitel J., Lippmann J., Hocke A.C., Zahlten J., Slevogt H., N’guessan P.D., Günther S., Schmeck B., Hippenstiel S. (2008). NAIP and Ipaf control Legionella pneumophila replication in human cells. J. Immunol..

[86] Zhao Y., Yang J., Shi J., Gong Y.N., Lu Q., Xu H., Liu L., Shao F. (2011). The NLRC4 inflammasome receptors for bacterial flagellin and type III secretion apparatus. Nature.

[87] Strowig T., Henao-Mejia J., Elinav E., Flavell R. (2012). Inflammasomes in health and disease. Nature.

[88] Petrasek J., Bala S., Csak T., Lippai D., Kodys K., Menashy V., Barrieau M., Min S.Y., Kurt-Jones E.A., Szabo G. (2012). IL-1 receptor antagonist ameliorates inflammasome-dependent alcoholic steatohepatitis in mice. J. Clin. Invest..

[89] Wree A., Eguchi A., McGeough M.D., Pena C.A., Johnson C.D., Canbay A., Hoffman H.M., Feldstein A.E. (2014). NLRP3 inflammasome activation results in hepatocyte pyroptosis, liver inflammation, and fibrosis in mice. Hepatology.

[90] Voican C.S., Njiké-Nakseu M., Boujedidi H., Barri-Ova N., Bouchet-Delbos L., Agostini H., Maitre S., Prévot S., Cassard-Doulcier A.M., Naveau S. (2015). Alcohol withdrawal alleviates adipose tissue inflammation in patients with alcoholic liver disease. Liver Int..

[91] Csak T., Ganz M., Pespisa J., Kodys K., Dolganiuc A., Szabo G. (2011). Fatty acid and endotoxin activate inflammasomes in mouse hepatocytes that release danger signals to stimulate immune cells. Hepatology.

[92] Gieling R.G., Wallace K., Han Y.P. (2009). Interleukin-1 participates in the progression from liver injury to fibrosis. Am. J. Physiol. Gastrointest Liver Physiol..

[93] Han Y., Chen Z., Hou R., Yan D., Liu C., Chen S., Li X., Du W. (2015). Expression of AIM2 is correlated with increased inflammation in chronic hepatitis B patients. Virol. J..

[94] Imaeda A.B., Watanabe A., Sohail M.A., Mahmood S., Mohamadnejad M., Sutterwala F.S., Flavell R.A., Mehal W.Z. (2009). Acetaminophen-induced hepatotoxicity in mice is dependent on Tlr9 and the Nalp3 inflammasome. J. Clin. Invest..

[95] Wei Q., Mu K., Li T., Zhang Y., Yang Z., Jia X., Zhao W., Huai W., Guo P., Han L. (2014). Deregulation of the NLRP3 inflammasome in hepatic parenchymal cells during liver cancer progression. Lab Invest..

[96] Fan S.H., Wang Y.Y., Lu J., Zheng Y.L., Wu D.M., Li M.Q., Hu B., Zhang Z.F., Cheng W., Shan Q. (2014). Luteoloside suppresses proliferation and metastasis of hepatocellular carcinoma cells by inhibition of NLRP3 inflammasome. PLoS ONE.

[97] Shiffman M.L., Pockros P., McHutchison J.G., Schiff E.R., Morris M., Burgess G. (2010). Clinical trial: The efficacy and safety of oral PF-03491390, a pancaspase inhibitor - a randomized placebo-controlled study in patients with chronic hepatitis C. Aliment. Pharmacol. Ther..

[98] Pockros P.J., Schiff E.R., Shiffman M.L., McHutchison J.G., Gish R.G., Afdhal N.H., Makhviladze M., Huyghe M., Hecht D., Oltersdorf T. (2007). Oral IDN-6556, an antiapoptotic caspase inhibitor, may lower aminotransferase activity in patients with chronic hepatitis C. Hepatology.

[99] MacKenzie S.H., Schipper J.L., Clark A.C. (2010). The potential for caspases in drug discovery. Curr. Opin. Drug Discov. Devel..

[100] Mangan M.S.J., Olhava E.J., Roush W.R., Seidel H.M., Glick G.D., Latz E. (2018). Targeting the NLRP3 inflammasome in inflammatory diseases. Nat. Rev. Drug Discov..

[101] Zhu P., Duan L., Chen J., Xiong A., Xu Q., Zhang H., Zheng F., Tan Z., Gong F., Fang M. (2011). Gene silencing of NALP3 protects against liver ischemia-reperfusion injury in mice. Hum. Gene. Ther..

[102] Jiménez-Castro M.B., Elias-Miró M., Casillas-Ramírez A., Peralta C., Abdeldayem H. (2012). Experimental Models in Liver Surgery. Hepatic Surgery.

[103] Fan C., Zwacka R.M., Engelhardt J.F. (1999). Therapeutic approaches for ischemia/reperfusion injury in the liver. J. Mol. Med..

[104] Okaya T., Lentsch A.B. (2005). Hepatic expression of S32A/S36A IkappaBalpha does not reduce postischemic liver injury. J. Surg. Res..

[105] Sonnenday C.J., Warren D.S., Cooke S.K., Dietz H.C., Montgomery R.A. (2004). A novel chimeric ribozyme vector produces potent inhibition of ICAM-1 expression on ischemic vascular endothelium. J. Gene. Med..

[106] Coito A.J., Buelow R., Shen X.D., Amersi F., Moore C., Volk H.D., Busuttil R.W., Kupiec-Weglinski J.W. (2002). Heme oxygenase-1 gene transfer inhibits inducible nitric oxide synthase expression and protects genetically fat Zucker rat livers from ischemia-reperfusion injury. Transplantation.

[107] Harada H., Wakabayashi G., Takayanagi A., Shimazu M., Matsumoto K., Obara H., Shimizu N., Kitajima M. (2002). Transfer of the interleukin-1 receptor antagonist gene into rat liver abrogates hepatic ischemia-reperfusion injury. Transplantation.

[108] Ke B., Lipshutz G.S., Kupiec-Weglinski J.W. (2006). Gene therapy in liver ischemia and reperfusion injury. Curr. Pharm. Des..

[109] Pachori A.S., Melo L.G., Hart M.L., Noiseux N., Zhang L., Morello F., Solomon S.D., Stahl G.L., Pratt R.E., Dzau V.J. (2004). Hypoxia-regulated therapeutic gene as a preemptive treatment strategy against ischemia/reperfusion tissue injury. Proc. Natl. Acad. Sci. USA.

[110] Somia N., Verma I.M. (2000). Gene therapy: Trials and tribulations. Nat. Rev. Genet..

[111] Inoue Y., Shirasuna K., Kimura H., Usui F., Kawashima A., Karasawa T., Tago K., Dezaki K., Nishimura S., Sagara J. (2014). NLRP3 regulates neutrophil functions and contributes to hepatic ischemia-reperfusion injury independently of inflammasomes. J. Immunol..

[112] Kamo N., Ke B., Ghaffari A.A., Shen X.D., Busuttil R.W., Cheng G., Kupiec-Weglinski J.W. (2013). ASC/caspase-1/IL-1β signaling triggers inflammatory responses by promoting HMGB1 induction in liver ischemia/reperfusion injury. Hepatology.

[113] Kato A., Gabay C., Okaya T., Lentsch A.B. (2002). Specific role of interleukin-1 in hepatic neutrophil recruitment after ischemia/reperfusion. Am. J. Pathol..

[114] Tan Z., Jiang R., Wang X., Wang Y., Lu L., Liu Q., Zheng S.G., Sun B., Ryffel B. (2013). RORγt+IL-17+ neutrophils play a critical role in hepatic ischemia-reperfusion injury. J. Mol. Cell Biol..

[115] Shito M., Wakabayashi G., Ueda M., Shimazu M., Shirasugi N., Endo M., Mukai M., Kitajima M. (1997). Interleukin 1 receptor blockade reduces tumor necrosis factor production, tissue injury, and mortality after hepatic ischemia-reperfusion in the rat. Transplantation.

[116] Takeuchi D., Yoshidome H., Kato A., Ito H., Kimura F., Shimizu H., Ohtsuka M., Morita Y., Miyazaki M. (2004). Interleukin 18 causes hepatic ischemia/reperfusion injury by suppressing anti-inflammatory cytokine expression in mice. Hepatology.

[117] Huang H., Chen H.W., Evankovich J., Yan W., Rosborough B.R., Nace G.W., Ding Q., Loughran P., Beer-Stolz D., Billiar T.R. (2013). Histones activate the NLRP3 inflammasome in Kupffer cells during sterile inflammatory liver injury. J. Immunol..

[118] Kim H.Y., Kim S.J., Lee S.M. (2015). Activation of NLRP3 and AIM2 inflammasomes in Kupffer cells in hepatic ischemia/reperfusion. FEBS J..

[119] Yue S., Zhu J., Zhang M., Li C., Zhou X., Zhou M., Ke M., Busuttil R.W., Ying Q.L., Kupiec-Weglinski J.W. (2016). The myeloid heat shock transcription factor 1/β-catenin axis regulates NLR family, pyrin domain-containing 3 inflammasome activation in mouse liver ischemia/reperfusion injury. Hepatology.

[120] Vargas R., Videla L.A. (2017). Thyroid hormone suppresses ischemia-reperfusion-induced liver NLRP3 inflammasome activation: Role of AMP-activated protein kinase. Immunol. Lett..

[121] Fernández V., Tapia G., Varela P., Castillo I., Mora C., Moya F., Orellana M., Videla L.A. (2005). Redox up-regulated expression of rat liver manganese superoxide dismutase and Bcl-2 by thyroid hormone is associated with inhibitor of kappaB-alpha phosphorylation and nuclear factor-kappaB activation. J. Endocrinol..

[122] Videla L.A., Cornejo P., Castillo I., Romanque P., Berhardt L.A. (2012). Thyroid hormone-induced regulatory interrelations in rat liver Nrf2-Keap1 signaling related toantioxidant enzyme expression. Advances in Medicine and Biology.

[123] Tapia G., Fernández V., Pino C., Ardiles L., Videla L.A. (2006). The acute-phaseresponse of the liver in relation to thyroid hormone-induced redox signalling. Free Radic. Biol. Med..

[124] Fernández V., Reyes S., Bravo S., Sepúlveda R., Romanque P., Santander G., Castillo I., Varela P., Tapia G., Videla L.A. (2007). Involvement of Kupffer cell-dependent signaling in T3-inducedhepatocyte proliferation in vivo. Biol. Chem..

[125] Hwang J.K., Yu H.N., Noh E.M., Kim J.M., Hong O.Y., Youn H.J., Jung S.H., Kwon K.B., Kim J.S., Lee Y.R. (2017). DHA blocks TPA-induced cell invasion by inhibiting MMP-9 expression via suppression of the PPAR-gamma/NF-kappaB pathway in MCF-7 cells. Oncol. Lett..

[126] Li Z., Zhao F., Cao Y., Zhang J., Shi P., Sun X., Zhang F., Tong L. (2018). DHA attenuates hepatic ischemia reperfusion injury by inhibiting pyroptosis and activating PI3K/Akt pathway. Eur. J. Pharmacol..

[127] Gaidt M.M., Hornung V. (2016). Pore formation by GSDMD is the effector mechanism of pyroptosis. EMBO J..

[128] Yaron J.R., Chen H., Ambadapadi S., Zhang L., Tafoya A.M., Munk B.H., Wakefield D.N., Fuentes J., Marques B.J., Harripersaud K. (2019). Serp-2, a virus-derived apoptosis and inflammasome inhibitor, attenuates liver ischemia-reperfusion injury in mice. J. Inflamm..

[129] Yang M., Antoine D.J., Weemhoff J.L., Jenkins R.E., Farhood A., Park B.K., Jaeschke H. (2014). Biomarkers distinguish apoptotic and necrotic cell death during hepatic ischemia/reperfusion injury in mice. Liver Transpl..

[130] Liu X., Hu Z., Zhou B., Li X., Tao R. (2015). Chinese Herbal Preparation Xuebijing Potently Inhibits Inflammasome Activation in Hepatocytes and Ameliorates Mouse Liver Ischemia-Reperfusion Injury. PLoS ONE.

[131] Mitchell J.R., Verweij M., Brand K., van de Ven M., Goemaere N., van den Engel S., Chu T., Forrer F., Müller C., de Jong M. (2010). Short-term dietary restriction and fasting precondition against ischemia reperfusion injury in mice. Aging Cell.

[132] Miyauchi T., Uchida Y., Kadono K., Hirao H., Kawasoe J., Watanabe T., Ueda S., Okajima H., Terajima H., Uemoto S. (2019). Up-regulation of FOXO1 and reduced inflammation by β-hydroxybutyric acid are essential diet restriction benefits against liver injury. Proc. Natl. Acad. Sci. USA.

[133] Verweij M., van Ginhoven T.M., Mitchell J.R., Sluiter W., van den Engel S., Roest H.P., Torabi E., Ijzermans J.N., Hoeijmakers J.H., de Bruin R.W. (2011). Preoperative fasting protects mice against hepatic ischemia/reperfusion injury: Mechanisms and effects on liver regeneration. Liver Transpl..

[134] Qin J., Zhou J., Dai X., Zhou H., Pan X., Wang X., Zhang F., Rao J., Lu L. (2016). Short-term starvation attenuates liver ischemia-reperfusion injury (IRI) by Sirt1-autophagy signaling in mice. Am. J. Transl. Res..

[135] Rickenbacher A., Jang J.H., Limani P., Ungethüm U., Lehmann K., Oberkofler C.E., Weber A., Graf R., Humar B., Clavien P.A. (2014). Fasting protects liver from ischemic injury through Sirt1-mediated downregulation of circulating HMGB1 in mice. J. Hepatol..

[136] Ando T., Ito H., Kanbe A., Hara A., Seishima M. (2017). Deficiency of NALP3 Signaling Impairs Liver Regeneration After Partial Hepatectomy. Inflammation.

[137] Cressman D.E., Greenbaum L.E., DeAngelis R.A., Ciliberto G., Furth E.E., Poli V., Taub R. (1996). Liver failure and defective hepatocyte regeneration in interleukin-6-deficient mice. Science.

[138] Yamada Y., Kirillova I., Peschon J.J., Fausto N. (1997). Initiation of liver growth by tumor necrosis factor: Deficient liver regeneration in mice lacking type I tumor necrosis factor receptor. Proc. Natl. Acad. Sci. USA.

[139] Böhm F., Köhler U.A., Speicher T., Werner S. (2010). Regulation of liver regeneration by growth factors and cytokines. EMBO Mol. Med..

[140] Lv M., Zeng H., He Y., Zhang J., Tan G. (2018). Dexmedetomidine promotes liver regeneration in mice after 70% partial hepatectomy by suppressing NLRP3 inflammasome not TLR4/NFκB. Int. Immunopharmacol..

[141] Yu S.X., Chen W., Hu X.Z., Feng S.Y., Li K.Y., Qi S., Lei Q.Q., Hu G.Q., Li N., Zhou F.H. (2017). Liver X receptors agonists suppress NLRP3 inflammasome activation. Cytokine.

[142] Mridha A.R., Wree A., Robertson A.A.B., Yeh M.M., Johnson C.D., Van Rooyen D.M., Haczeyni F., Teoh N.C., Savard C., Ioannou G.N. (2017). NLRP3 inflammasome blockade reduces liver inflammation and fibrosis in experimental NASH in mice. J. Hepatol..

[143] DeSantis D.A., Ko C.W., Wang L., Lee P., Croniger C.M. (2015). Constitutive Activation of the Nlrc4 Inflammasome Prevents Hepatic Fibrosis and Promotes Hepatic Regeneration after Partial Hepatectomy. Mediators Inflamm..

[144] Casillas-Ramírez A., Escobedo-Medina S.G., Cordero-Pérez P., Jiménez-Castro M.B., Peralta C., Gracia-Gracia J., Salvadó J. (2016). Ischemia-reperfusion injury and oxidative stress. Gastrointestinal Tissue: Oxidative Stress and Dietary Antioxidants.

[145] Liu H., Lo C.M., Yeung O.W.H., Li C.X., Liu X.B., Qi X., Ng K.T.P., Liu J., Ma Y.Y., Lam Y.F. (2017). NLRP3 inflammasome induced liver graft injury through activation of telomere-independent RAP1/KC axis. J. Pathol..

[146] Ling C.C., Ng K.T., Shao Y., Geng W., Xiao J.W., Liu H., Li C.X., Liu X.B., Ma Y.Y., Yeung W.H. (2014). Post-transplant endothelial progenitor cell mobilization via CXCL10/CXCR3 signaling promotes liver tumor growth. J. Hepatol..

[147] Sonohara F., Inokawa Y., Kanda M., Nishikawa Y., Yamada S., Fujii T., Sugimoto H., Kodera Y., Nomoto S. (2017). Association of Inflammasome Components in Background Liver with Poor Prognosis After Curatively-resected Hepatocellular Carcinoma. Anticancer Res..

[148] He W., Ye S., Zeng C., Xue S., Hu X., Zhang X., Gao S., Xiong Y., He X., Vivalda S. (2018). Hypothermic oxygenated perfusion (HOPE) attenuates ischemia/reperfusion injury in the liver through inhibition of the TXNIP/NLRP3 inflammasome pathway in a rat model of donation after cardiac death. FASEB J..

[149] Schlegel A., Kron P., Dutkowski P. (2016). Hypothermic machine perfusion in liver transplantation. Curr. Opin. Organ. Transplant.

[150] Li P., Liu Y.F., Yang L. (2015). Advantages of dual hypothermic oxygenated machine perfusion over simple cold storage in the preservation of liver from porcine donors after cardiac death. Clin Transplant.

[151] Westerkamp A.C., Karimian N., Matton A.P., Mahboub P., van Rijn R., Wiersema-Buist J., de Boer M.T., Leuvenink H.G., Gouw A.S., Lisman T. (2016). Oxygenated hypothermic machine perfusion after static cold storage improves hepatobiliary functionof extendedcriteriadonorlivers. Transplantation.

[152] Hong B.J., Liu H., Wang Z.H., Zhu Y.X., Su L.Y., Zhang M.X., Xu K., Chen J.Z. (2017). Inflammasome activation involved in early inflammation reaction after liver transplantation. Immunol. Lett..

[153] Li X., Wu Y., Zhang W., Gong J., Cheng Y. (2017). Pre-conditioning with tanshinone IIA attenuates the ischemia/reperfusion injury caused by liver grafts via regulation of HMGB1 in rat Kupffer cells. Biomed. Pharmacother..

[154] Sadowsky D., Zamora R., Barclay D., Yin J., Fontes P., Vodovotz Y. (2016). Machine Perfusion of Porcine Livers with Oxygen-Carrying Solution Results in Reprogramming of Dynamic Inflammation Networks. Front Pharmacol..

[155] Yu Y., Cheng Y., Pan Q., Zhang Y.J., Jia D.G., Liu Y.F. (2019). Effect of the Selective NLRP3 Inflammasome Inhibitor mcc950 on Transplantation Outcome in a Pig Liver Transplantation Model with Organs From Donors After Circulatory Death Preserved by Hypothermic Machine Perfusion. Transplantation.

[156] Karp S.J., Johnson S., Evenson A., Curry M.P., Manning D., Malik R., Lake-Bakaar G., Lai M., Hanto D. (2011). Minimising cold ischaemic time is essential in cardiac death donor-associated liver transplantation. HPB Oxford.

[157] Smith M., Dominguez-Gil B., Greer D.M., Manara A.R., Souter M.J. (2019). Organ donation after circulatory death: Current status and future potential. Intensive Care Med..

[158] Tang J.X., Na N., Li J.J., Fan L., Weng R.H., Jiang N. (2018). Outcomes of Controlled Donation After Cardiac Death Compared with Donation After Brain Death in Liver Transplantation: A Systematic Review and Meta-analysis. Transplant Proc..

[159] Selzner M., Clavien P.A. (2001). Fatty liver in liver transplantation and surgery. Semin. Liver Dis..

[160] Zhong L., Li H., Li Z., Shi B., Wang P., Wang C., Fan J., Sun H., Wang P., Qin X. (2016). C7 genotype of the donor may predict early bacterial infection after liver transplantation. Sci. Rep..

[161] Baccarani U., Isola M., Adani G.L., Avellini C., Lorenzin D., Rossetto A., Currò G., Comuzzi C., Toniutto P., Risaliti A. (2010). Steatosis of the hepatic graft as a risk factor for post-transplant biliary complications. Clin. Transplant.

